# Nanoparticle-Mediated Radiosensitization in Breast Cancer: A Systematic Review of Preclinical Evidence and Translational Challenges

**DOI:** 10.3390/ijms27146522

**Published:** 2026-07-22

**Authors:** Sorinel Lunca, Stefan Morarasu, Gabriel Mihail Dimofte

**Affiliations:** 1 Grigore T Popa University of Medicine and Pharmacy, 700115 Iasi, Romania; sorinel.lunca@umfiasi.ro (S.L.); mihail.dimofte@umfiasi.ro (G.M.D.); 2 2nd Department of Surgical Oncology, Regional Institute of Oncology, 700483 Iasi, Romania

**Keywords:** breast cancer, radiosensitization, nanoparticles, radiotherapy, tumor microenvironment, nanomedicine, triple-negative breast cancer

## Abstract

Radiotherapy is a cornerstone of breast cancer treatment, but its efficacy is frequently limited by intrinsic and acquired radioresistance as well as dose-limiting toxicity to surrounding normal tissues. Nanoparticle-mediated radiosensitization has emerged as a promising strategy to enhance the therapeutic index of irradiation by combining physical dose amplification with biological, microenvironmental, and immunological modulation. In this systematic review, we evaluated preclinical evidence on nanoparticle-mediated radiosensitization in breast cancer, with emphasis on nanoplatform design, mechanistic patterns, therapeutic efficacy, and translational relevance. A total of 66 studies published between 2015 and 2026 were included. The identified systems encompassed a broad range of materials, including gold-, silver-, platinum-, bismuth-, gadolinium-, polymer-, lipid-, and hybrid-based nanoplatforms, frequently incorporating targeting ligands, catalytic components, biomimetic coatings, or therapeutic payloads. Enhanced radiation responses were most commonly associated with high-atomic-number (high-Z)-mediated energy deposition, increased reactive oxygen species generation, and enhanced DNA damage persistence. Additional mechanisms, including redox modulation, hypoxia targeting, regulated cell death, and immune activation, reflect the evolution of nanoparticle-assisted radiotherapy from predominantly physical radioenhancement toward multifunctional physicobiological strategies. Triple-negative breast cancer models predominated throughout the literature. Across preclinical models, nanoparticle-assisted irradiation consistently improved clonogenic survival, tumor control, and, in selected studies, survival. However, substantial heterogeneity in study design and limited use of rigorous radiobiological endpoints restricted cross-study comparability. The available preclinical evidence indicates that the most promising nanoparticle-mediated radiosensitization strategies integrate physical dose enhancement with biologically active mechanisms targeting oxidative stress, hypoxia, persistent DNA damage, immune signaling, and tumor microenvironmental resistance. Collectively, these findings suggest that the field is evolving from predominantly physical radioenhancement toward multifunctional, mechanism-driven physicobiological strategies. However, clinical translation remains constrained by methodological heterogeneity and limited radiobiological validation, highlighting the need for standardized preclinical evaluation and clinically feasible nanoplatforms tailored to subtype-specific mechanisms of radioresistance.

## 1. Introduction

Breast cancer remains the most commonly diagnosed malignancy worldwide and one of the leading causes of cancer-related mortality among women. According to GLOBOCAN 2022 estimates, breast cancer accounted for approximately 11.6% of all newly diagnosed cancer cases and 6.9% of all cancer-related deaths worldwide, corresponding to an estimated 2.3 million new cases and 670,000 deaths annually [1,2].

Despite significant advances in systemic therapies, including targeted agents and immunotherapy, radiotherapy remains a cornerstone of multidisciplinary breast cancer management and is widely used in both curative and palliative settings. In addition to improving local control following breast-conserving surgery, radiotherapy contributes to survival benefit in selected high-risk patients [3]. However, its efficacy is frequently limited by intrinsic and acquired radioresistance, as well as by the risk of damage to surrounding normal tissues, particularly in anatomically sensitive regions such as the heart and lungs [3,4,5].

Radioresistance in breast cancer is a multifactorial process involving tumor cell-intrinsic mechanisms, including enhanced DNA damage repair capacity, dysregulated cell cycle checkpoints, and resistance to apoptosis, as well as extrinsic factors related to the tumor microenvironment [4]. Hypoxia is one of the most important determinants of radiotherapy response because reduced oxygen availability limits stabilization of radiation-induced DNA damage and decreases sensitivity to ionizing radiation [6]. In addition, stromal components such as cancer-associated fibroblasts (CAFs), immune suppressive cells, and extracellular matrix remodeling contribute to treatment resistance by promoting survival signaling, enhancing DNA repair, and limiting drug and oxygen delivery [2,7]. These challenges are particularly pronounced in aggressive subtypes such as triple-negative breast cancer (TNBC), which is characterized by high genomic instability, lack of actionable receptors, and poor clinical outcomes [8].

In this context, radiosensitization has emerged as a key strategy to enhance the therapeutic ratio of radiotherapy by increasing tumor sensitivity while minimizing normal tissue toxicity [4,9]. Conventional radiosensitizers, including chemotherapeutic agents such as cisplatin or gemcitabine, have shown clinical utility but are often associated with systemic toxicity and limited tumor specificity [3,5]. Consequently, there is a growing interest in developing more selective and effective radiosensitizing approaches that can overcome biological resistance mechanisms without exacerbating treatment-related side effects.

Nanotechnology has emerged as a promising strategy for enhancing radiation response because nanoparticles can be engineered to promote both physical dose amplification and biological modulation, thereby increasing the potential for radiosensitization [9,10]. Nanoparticles can be engineered with precise control over size, shape, composition, and surface functionalization, enabling tailored interactions with biological systems and selective accumulation in tumor tissues [11,12]. One of the fundamental principles underlying nanoparticle-mediated radiosensitization is the use of high-atomic-number (high-Z) materials, such as gold, hafnium, bismuth, and gadolinium, which exhibit increased photoelectric absorption and generate secondary electrons under irradiation, thereby amplifying local radiation dose deposition [9,13].

Beyond physical dose enhancement, nanoparticles can also modulate a wide range of biological processes relevant to radiation response. These include the generation of reactive oxygen species (ROS), depletion of intracellular antioxidants, inhibition of DNA repair pathways, and induction of alternative cell death mechanisms such as ferroptosis and pyroptosis [14,15,16,17]. Importantly, nanoparticle systems can be designed to address key features of the tumor microenvironment, such as hypoxia, by delivering oxygen, catalyzing endogenous reactions, or remodeling stromal and immune components [17,18,19]. These integrated capabilities distinguish nanoparticle-based radiosensitizers from conventional agents and provide opportunities for synergistic therapeutic effects.

Recent advances in nanomedicine have further expanded the scope of radiosensitization by integrating targeting ligands, drug delivery systems, and immune-modulating components into integrated nanoplatforms [20,21,22,23]. Targeted nanoparticles can preferentially accumulate in tumor cells through receptor-mediated mechanisms, such as HER2 or integrin targeting, thereby increasing treatment specificity and reducing off-target effects [20,24]. HER2 (human epidermal growth factor receptor 2), encoded by the *ERBB2* oncogene, is overexpressed in approximately 15–20% of breast cancers and represents a clinically validated therapeutic target. In contrast, triple-negative breast cancer (TNBC) lacks HER2 expression and therefore requires alternative receptor-targeting or microenvironment-targeting approaches for selective nanoparticle delivery [20,24]. In parallel, biomimetic and cell membrane-coated nanoparticles have been developed to enhance circulation time, evade immune clearance, and improve tumor penetration [22,25]. Moreover, the combination of radiosensitization with immunotherapy has gained increasing attention, as radiotherapy can induce immunogenic cell death and stimulate systemic antitumor immune responses [23,26].

Despite these promising developments, the translation of nanoparticle radiosensitizers into clinical practice remains limited. Challenges include variability in nanoparticle biodistribution, limited tumor penetration, potential long-term toxicity, and difficulties in large-scale manufacturing and regulatory approval [10,11,12]. Furthermore, the heterogeneity of preclinical models and the lack of standardized experimental protocols complicate the interpretation and comparison of results across studies. These issues highlight the need for systematic evaluation of the existing evidence to identify key trends, mechanisms, and gaps in the field.

In breast cancer, nanoparticle-mediated radiosensitization represents a particularly attractive therapeutic strategy because radiotherapy plays a central role in disease management, yet treatment response varies substantially among molecular subtypes and is influenced by multiple biological determinants of radioresistance [3]. Tumor hypoxia, enhanced DNA repair capacity, oxidative stress adaptation, immune-suppressive tumor microenvironments, and cancer stem cell populations all contribute to reduced radiation efficacy [4,5,6,7,8]. These characteristics provide multiple opportunities for nanoparticle-mediated intervention through enhanced local energy deposition, ROS amplification, redox modulation, hypoxia alleviation, and immune activation. The biological heterogeneity of breast cancer further strengthens its relevance as a model for nanoparticle-assisted radiotherapy. Triple-negative breast cancer (TNBC), in particular, is characterized by genomic instability, hypoxic microenvironments, dysregulated DNA repair pathways, and limited therapeutic options, making it especially suitable for advanced radiosensitization strategies [8,10]. In addition, the accessibility of breast tumors for imaging and image-guided treatment supports the development of theranostic nanoplatforms capable of integrating diagnosis, treatment delivery, and response monitoring.

Despite these advances, preclinical evidence on nanoparticle-mediated radiosensitization in breast cancer remains highly heterogeneous, with substantial variability in nanoplatform design, experimental models, irradiation protocols, and reported outcomes. Moreover, a structured synthesis linking nanoparticle design features with radiosensitization mechanisms and therapeutic responses in breast cancer is lacking. Therefore, the present systematic review evaluates how nanoparticle composition and functionalization strategies influence radiation-associated biological responses, radiosensitization-related mechanisms, and therapeutic efficacy in preclinical breast cancer models. In addition to descriptive synthesis, this review critically examines methodological heterogeneity, translational limitations, and current gaps in the field, including the lack of standardized experimental frameworks and limited comparative analyses across breast cancer subtypes. By integrating nanoplatform characteristics with underlying biological mechanisms and therapeutic outcomes, this work aims to provide a structured framework to support the rational development and translational advancement of nanoparticle-based radiosensitization strategies.

## 2. Materials and Methods

### 2.1. Search Strategy

This systematic review was conducted in accordance with the Preferred Reporting Items for Systematic Reviews and Meta-Analyses (PRISMA) guidelines (see Supplementary Materials PRISMA 2020 Checklist) and was prospectively registered in the International Prospective Register of Systematic Reviews (PROSPERO; registration number CRD420261365298).

A comprehensive literature search was conducted in MEDLINE (via PubMed) and EMBASE (Elsevier, Amsterdam, The Netherlands, https://www.embase.com/) to identify relevant studies published between 1 January 2015 and 2 March 2026. MEDLINE and EMBASE were selected as the primary databases due to their comprehensive coverage of biomedical and translational research, including oncology, radiotherapy, and nanomedicine literature. Additional databases were not included to maintain consistency and feasibility of systematic screening. The search strategy combined controlled vocabulary terms (MeSH and Emtree) with free-text keywords related to breast cancer, radiotherapy, radiosensitization, and nanotechnology. Representative search terms included “breast cancer”, “breast neoplasm”, “mammary carcinoma”, “radiotherapy”, “radiation therapy”, “radiosensitization”, “radiosensitizer”, “nanoparticle”, “nanomaterial”, “nanotechnology”, “gold nanoparticle”, and “metal-organic framework”. Search terms were adapted appropriately for each database, and the complete database-specific search strategies including controlled vocabulary terms, free-text keywords, Boolean operators, and filters, are provided in Supplementary Table S1.

The final database searches were conducted on 2 March 2026. To ensure inclusion of primary experimental evidence, filters were applied to exclude reviews, editorials, letters, and meta-analyses. The search was limited to English-language publications. In EMBASE, additional filters were applied to restrict results to preclinical studies, including in vitro, in vivo, animal experiments, and cell culture studies.

### 2.2. Eligibility Criteria

Studies were considered eligible if they investigated nanoparticle-based radiosensitization in breast cancer models, included radiotherapy or irradiation as part of the experimental design, and were conducted in preclinical settings. Studies were excluded if they were clinical investigations involving human subjects, did not evaluate nanoparticle-mediated radiosensitization, did not include irradiation, focused on non-breast cancer models, or were non-original articles (reviews, editorials, letters, or conference abstracts).

### 2.3. Study Selection

All identified records were exported into reference management software (Rayyan software version 1.4.3. 2024) and duplicates were removed prior to screening. Title and abstract screening was performed independently by two reviewers. Full-text articles of potentially eligible studies were subsequently assessed independently by the same reviewers. Disagreements were resolved by consensus, and when consensus could not be reached, a third reviewer acted as arbitrator.

### 2.4. Data Extraction

Data extraction was performed using a predefined standardized framework. Two reviewers independently extracted data from each included study, and the results were subsequently compared and reconciled by consensus.

Extracted variables included nanoparticle characteristics, experimental design, radiotherapy parameters, mechanistic endpoints, and therapeutic outcomes. Nanoparticle-related variables comprised material composition, particle size, surface modification, targeting strategies, and incorporated therapeutic or diagnostic payloads. Experimental variables included breast cancer subtype, cell lines, animal models, and overall study design. Radiotherapy-related variables included radiation type, dose, and treatment schedule when reported.

Mechanistic endpoints included reactive oxygen species generation, DNA damage, hypoxia modulation, regulated cell death pathways such as apoptosis, ferroptosis, and pyroptosis, immune activation, and nanoparticle-mediated delivery or microenvironmental effects. Therapeutic endpoints included changes in cell viability, clonogenic survival, tumor growth inhibition, survival outcomes, and radiosensitization metrics such as sensitizer enhancement ratio or dose enhancement factor when available.

### 2.5. Classification of Radiosensitization Mechanisms

To enable structured synthesis, mechanistic categories were assigned based on reported experimental findings, including reactive oxygen species (ROS) generation, DNA damage, hypoxia modulation, regulated cell death pathways, and immune activation.

Mechanistic classification was based on the level of experimental evidence reported by each study, and categories were interpreted as follows: “+” (present): direct experimental evidence supporting the involvement of a given mechanism, based on quantitative assays or pathway-specific analyses (e.g., ROS measurement assays, γ-H2AX staining, hypoxia markers, expression of pathway-related proteins); “±” (partial/indirect): suggestive or secondary evidence, including indirect markers, correlative findings, or incomplete pathway evaluation; “−” (absent): no evidence reported for the given mechanism.

These classifications reflect reported associations rather than definitive causal relationships, as most studies did not include mechanistic inhibition or rescue experiments to confirm pathway-specific contributions.

### 2.6. Data Synthesis

Given the substantial heterogeneity in nanoparticle platforms, physicochemical design, biological functionalization, experimental models, irradiation protocols, and outcome measures, a formal meta-analysis was not performed. Instead, the data were synthesized qualitatively, with results organized according to nanoplatform design and functionalization strategies, mechanisms of radiation-associated biological enhancement, and therapeutic outcomes. For the purposes of this review, radiosensitization was defined as a quantitatively demonstrated increase in radiation effect attributable to nanoparticle intervention, supported by validated radiobiological endpoints such as clonogenic survival assays, sensitizer enhancement ratio (SER), dose enhancement factor (DEF), dose-modifying factor (DMF), or equivalent dose–response analyses. In contrast, biological effects such as increased ROS generation, enhanced DNA damage, apoptosis, tumor growth inhibition, or survival benefit were interpreted as evidence of radiation-associated enhancement of therapeutic activity unless accompanied by formal radiobiological validation. Accordingly, radiosensitization was distinguished from radioenhancement throughout the review whenever sufficient methodological information was available.

### 2.7. Study Quality Assessment

A structured qualitative assessment of methodological quality was conducted for all included studies using a predefined framework tailored to preclinical nanomedicine research, adapted from commonly reported quality domains in experimental oncology. Six domains were evaluated: nanoparticle characterization, experimental model relevance, radiotherapy protocol reporting, outcome assessment rigor, mechanistic depth, and internal validity safeguards. Each domain was rated as high, moderate, or low based on completeness of reporting and methodological rigor.

Nanoparticle characterization was assessed based on the extent of physicochemical reporting, including particle size, morphology, surface charge, composition, stability, and reproducibility, with high ratings assigned to studies providing comprehensive characterization across multiple parameters, moderate ratings to those with partial reporting, and low ratings to studies with minimal or absent characterization. Experimental model relevance was evaluated according to the biological and translational appropriateness of the models used, with high-quality studies incorporating clinically relevant models such as orthotopic, metastatic, or multiple complementary systems, moderate-quality studies relying on standard in vitro or in vivo models, and low-quality studies using limited or poorly justified models.

Radiotherapy protocol reporting was assessed based on the level of detail provided regarding irradiation parameters, including radiation type, dose, fractionation, and treatment conditions. Studies with comprehensive reporting were rated as high, those with partial information as moderate, and those lacking essential details as low. Outcome assessment rigor was evaluated according to the use of validated and quantitative endpoints, such as clonogenic survival assays, survival analysis, and standardized biomarkers, with lower ratings assigned to studies relying on limited or non-standard measures.

Mechanistic depth was assessed based on the extent of mechanistic investigation, with high ratings assigned to studies demonstrating multi-level validation through molecular assays, pathway analyses, and functional experiments, moderate ratings to those with limited mechanistic exploration, and low ratings to studies providing only descriptive or indirect evidence. Finally, internal validity safeguards were evaluated based on the presence of appropriate controls, experimental reproducibility, and clarity of study design, with high-quality studies demonstrating robust experimental rigor and lower ratings assigned to studies with incomplete reporting or unclear methodological approaches.

Overall study quality was determined through an integrated qualitative appraisal across all domains. Given the heterogeneity of study designs and the preclinical nature of the included evidence, no formal quantitative risk-of-bias tool was applied. Quality assessment was performed independently by two reviewers, with discrepancies resolved by consensus. The results of the quality assessment are presented in Supplementary Table S2. A generative artificial intelligence (GenAI) tool, Canva Magic Design (Canva Pty Ltd., Sydney, Australia, Magic Design™: Free Online AI Design Tool | Canva, accessed on 30 May 2026), was used to assist in the visual design and graphical organization of Figure 2, which illustrates the mechanisms of nanoparticle-mediated radiosensitization in breast cancer.

## 3. Results

### 3.1. Study Selection

Records identified through database searching were exported and merged using reference management software, and duplicates were removed prior to screening. A total of 1905 records were identified, including 712 from MEDLINE and 1193 from EMBASE. After removal of 393 duplicates, 1512 records remained for title and abstract screening.

During this stage, 1313 records were excluded due to lack of relevance, including absence of radiotherapy-related content, non-breast cancer focus, lack of nanoparticle-based approaches, absence of radiosensitization concepts, or non-preclinical study design. A total of 199 full-text articles were assessed for eligibility.

Of these, 133 studies were excluded for predefined reasons, including lack of a true radiosensitization assessment (n = 42), nanoparticles not evaluated as the primary radiosensitizing intervention (n = 31), use of non-breast cancer models (n = 21), insufficient or unusable data (n = 27), or other reasons (n = 12).

**Figure 1:**
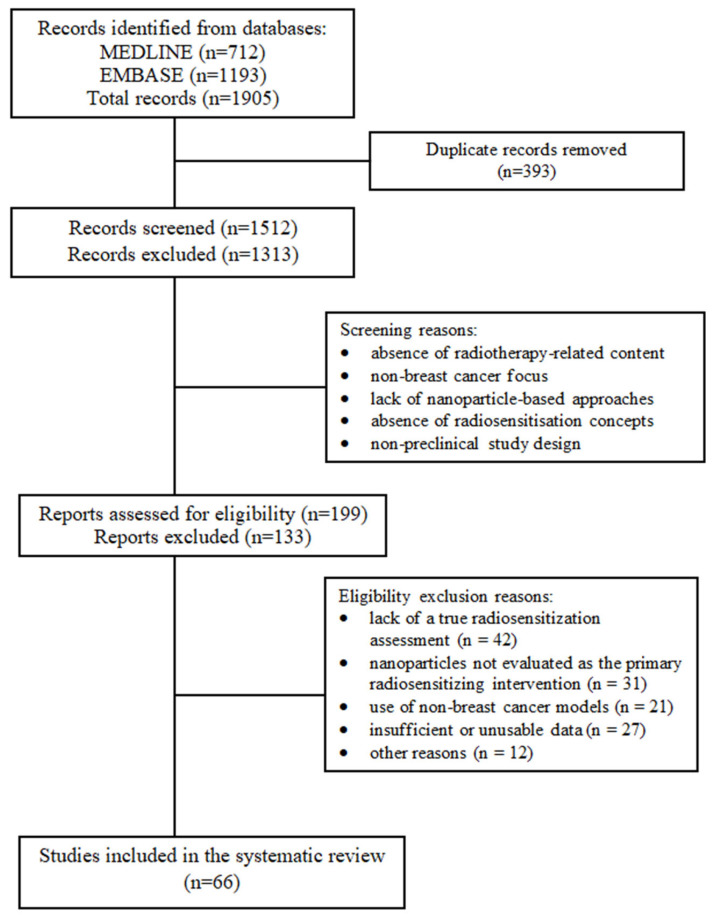
PRISMA 2020 flow diagram illustrating the process of study identification, screening, eligibility assessment, and inclusion.

Ultimately, 66 studies met all inclusion criteria and were included in the qualitative synthesis. The study selection process is summarized in the PRISMA 2020 flow diagram (Figure 1).

### 3.2. Quantitative Overview of the Included Studies

To provide an overview of the evidence base, key quantitative characteristics of the included studies are summarized below. Among the 66 included studies, triple-negative breast cancer (TNBC) was the predominant experimental model, represented in 39 of 66 studies (59%), whereas HER2-positive models were used in 4 studies (6%) and luminal models in 1 study (2%). Mixed or multiple-subtype models accounted for 6 studies (9%), while 16 studies (24%) used general or unspecified breast cancer models.

From an experimental design perspective, the majority of studies (44/66, 67%) combined both in vitro and in vivo approaches, while 16 studies (24%) were limited to in vitro models and 6 studies (9%) were conducted exclusively in vivo.

Regarding radiosensitization assessment, clonogenic survival assays were reported in 26 of 66 studies (39%), whereas formal radiosensitization metrics such as sensitizer enhancement ratio (SER) or dose enhancement factor (DEF) were used in 5 studies (8%). The limited use of clonogenic survival assays and quantitative radiosensitization metrics indicates that formal radiobiological validation was absent in a substantial proportion of studies despite widespread reporting of enhanced therapeutic activity following nanoparticle-assisted irradiation.

Evaluation of survival outcomes was performed in 5 studies (8%), and toxicity or safety assessments were reported in 22 studies (33%).

**Figure 2:**
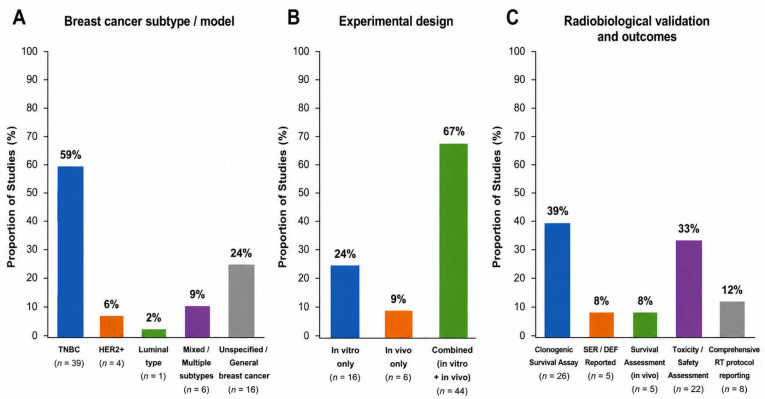
Quantitative overview of the included studies and radiobiological validation endpoints. (**A**) Distribution of breast cancer molecular subtypes represented in the 66 included studies. (**B**) Distribution of experimental designs according to the use of in vitro, in vivo, or combined in vitro/in vivo models. (**C**) Frequency of key radiobiological validation endpoints, including clonogenic survival assays, formal radiosensitization metrics (sensitizer enhancement ratio [SER] or dose enhancement factor [DEF]), and survival assessment. The figure highlights the predominance of triple-negative breast cancer (TNBC) models, the widespread use of combined experimental designs, and the limited use of gold-standard radiobiological validation endpoints across the included literature.

Radiotherapy protocol reporting was frequently incomplete, with only 8 of 66 studies (12%) providing comprehensive details on radiation type, dose, and treatment schedule, highlighting a major limitation in experimental standardization (Figure 2).

### 3.3. Characteristics of Nanoplatforms, Experimental Models, and Radiotherapy Protocols

Beyond the quantitative overview presented above, the included studies exhibited substantial diversity in nanoplatform composition, functionalization strategies, experimental models, and radiotherapy protocols.

A total of 66 preclinical studies investigating nanoparticle-mediated radiosensitization in breast cancer were included in the qualitative synthesis (Table 1, Table 2 and Table 3; Supplementary Tables S3–S5). The studies, published between 2015 and early 2026, exhibited substantial heterogeneity in nanomaterial composition, functionalization strategies, experimental models, and radiotherapy protocols [15,16,17,19,20,21,22,24,27,28,29,30,31,32,33,34,35,36,37,38,39,40,41,42,43,44,45,46,47,48,49,50,51,52,53,54,55,56,57,58,59,60,61,62,63,64,65,66,67,68,69,70,71,72,73,74,75,76,77,78,79,80,81,82,83,84].

Across the included studies, a wide spectrum of nanoplatforms was identified, with a predominance of metallic and hybrid high-atomic-number systems. Gold-based nanoparticles were the most frequently investigated class [20,24,27,28,29], followed by silver-, platinum-, bismuth-, and gadolinium-based platforms [15,30,31,32,33,34,35,36]. Hybrid and composite nanostructures, including metal–organic frameworks, organosilica systems, and multifunctional nanoplatforms integrating catalytic, magnetic, or imaging capabilities, were also reported [17,37,38,39,40,41]. Lipid-based and polymeric nanoparticles were represented in studies focusing on drug delivery, metabolic modulation, and tumor microenvironment targeting [19,42,43,44,45,46,47,48,49,50].

A high degree of functionalization was reported across studies. Many nanoplatforms incorporated targeting ligands, therapeutic payloads, and microenvironment-responsive components [16,17,26,27,45,49,51,52,53]. Receptor-specific targeting approaches, including HER2-, CXCR4-, and folate-directed systems, were described in association with increased cellular uptake [20,24,29,51,54,55]. Biomimetic strategies, including cell membrane-coated nanoparticles, were reported to enhance circulation time and immune evasion [34,44,50,56]. Catalytic and microenvironment-responsive systems, particularly MnO_2_-based platforms, were also described [17,19,21,53]. Therapeutic payload delivery was reported across multiple studies, including chemotherapeutic agents, epigenetic modulators, and nucleic acid-based therapeutics [16,27,42,45,52].

From an experimental standpoint, most studies employed both in vitro and in vivo approaches [17,20,27,39,54], whereas a smaller subset relied exclusively on a single model type [30,57,58]. Triple-negative breast cancer (TNBC) was the most frequently used experimental model [17,26,39,40,45], with frequent use of MDA-MB-231 cells and murine 4T1 models [17,20,27,39,40,45,54]. HER2-positive and luminal subtypes were less frequently investigated [29,51,57,59,60], and a limited number of studies included multiple subtypes within the same experimental design [28,57]. More complex experimental settings, including orthotopic, metastatic, and dual-tumor models, were reported in a subset of studies [17,22,43]. The principal characteristics of all included studies, including nanoplatform composition, functionalization strategies, experimental models, and breast cancer subtype representation, are summarized in Table 1.

Radiotherapy protocols varied across studies. X-ray irradiation was the most frequently reported modality [17,20,27,34,54], whereas megavoltage radiation and radionuclide-based approaches were less commonly used [20,24,61,62]. Reporting of radiation dose, fractionation, and treatment schedules was often incomplete. Variability in nanoparticle design, functionalization, and experimental parameters was observed across studies (Table 1, Table 2 and Table 3).

### 3.4. Nanoplatform Design and Functionalization Strategies

#### 3.4.1. High-Z Nanoplatforms

High-atomic-number (high-Z) nanoplatforms based on silver (Z = 47), gadolinium (Z = 64), platinum (Z = 78), gold (Z = 79), and bismuth (Z = 83) enhance radiation-induced energy deposition through photoelectric interactions and secondary electron generation [15,20,27,28,30,33,34,36,50,63]. Increased ROS production and DNA damage following irradiation were commonly reported in association with these systems [15,20,27,30,33], as summarized in Table 2.

Early studies predominantly investigated relatively simple high-Z nanostructures designed primarily to maximize physical dose enhancement through radiation energy deposition [30,64,65]. As the field evolved, increasing emphasis was placed on functional modifications, including surface coatings, targeting ligands, biomimetic components, and therapeutic payloads, giving rise to multifunctional nanoplatforms capable of improving cellular uptake and integrating biological mechanisms of radiosensitization [24,27,29,55,66]. Collectively, these advances reflect the progressive evolution of nanoparticle-assisted radiotherapy from predominantly physical radioenhancement toward integrated physicobiological strategies.

#### 3.4.2. Hybrid and Multifunctional Systems

Hybrid and integrated nanoplatforms were widely reported, integrating multiple components within a single system. These platforms included metal–organic frameworks, organosilica nanoparticles, and composite structures combining metallic cores with catalytic, magnetic, or imaging elements [17,37,38,39,40,41].

Such systems frequently incorporated catalytic and microenvironment-responsive components designed to address tumor hypoxia, redox imbalance, and metabolic vulnerabilities within the tumor microenvironment [17,19,21,43,47,53].

Multimodal therapeutic approaches were also described, combining radiotherapy with chemotherapy, photothermal therapy, photodynamic therapy, or immunomodulatory strategies [39,43,49,50,67,68,69]. These features were reported within integrated nanoplatform designs [17,39,40,41,68], as summarized in Table 2 and Supplementary Tables S3 and S4.

#### 3.4.3. Targeting and Functionalization Strategies

Targeting and functionalization strategies were reported across a large proportion of studies. These included receptor-mediated targeting using antibodies, peptides, and small molecules, as well as biomimetic approaches based on cell membrane coatings.

Receptor-specific targeting, including HER2-, CXCR4-, and folate-directed systems, was frequently described and associated with increased cellular uptake [20,24,29,49,51,55,70]. Biomimetic strategies, particularly cell membrane-coated nanoparticles, were reported to enhance circulation and immune evasion [21,34,44].

Therapeutic payload delivery was also commonly described, including chemotherapeutic agents, epigenetic modulators, and nucleic acid-based therapeutics [27,42,45,52,71,72,73]. Microenvironment-responsive functionalization targeting hypoxia, pH, and redox conditions was reported in multiple studies [17,19,53,67].

### 3.5. Radiosensitization Mechanisms

A quantitative overview of the reported mechanistic endpoints showed that ROS generation and DNA damage enhancement were the predominant biological responses investigated across the included studies. ROS-related effects were reported in 63 of 66 studies (95.5%), whereas enhanced DNA damage was described in 50 studies (75.8%). Regulated cell death pathways were investigated in 32 studies (48.5%), most commonly apoptosis (23/66, 34.8%), while ferroptosis (5/66, 7.6%), immunogenic cell death (3/66, 4.5%), and pyroptosis (2/66, 3.0%) were reported less frequently. Hypoxia modulation and immune activation were described in 10 (15.2%) and 11 (16.7%) studies, respectively. Overall, the current evidence base is dominated by oxidative stress- and DNA damage-related mechanisms, whereas tumor microenvironment modulation and immune-related mechanisms have emerged more recently, primarily within multifunctional nanoplatforms.

#### 3.5.1. Physical Dose Enhancement and ROS Generation

Physical dose enhancement and reactive oxygen species (ROS) generation were the most frequently reported mechanisms contributing to nanoparticle-mediated radiotherapy enhancement. High-atomic-number (high-Z) nanomaterials were reported to increase local radiation energy deposition through photoelectric interactions, resulting in the generation of secondary electrons and localized dose amplification [15,20,27,28,34]. In general, materials with higher atomic numbers exhibit greater photoelectric interaction probabilities and therefore greater potential for secondary electron generation and local dose enhancement, particularly at kilovoltage photon energies [9,10,84,85,86]. However, the overall magnitude of radiotherapy enhancement is not determined by atomic number alone but is also influenced by nanoparticle composition, size, surface functionalization, intracellular localization, and other physicochemical and biological characteristics [17,21,31,87].

This process was consistently associated with increased ROS production following irradiation, leading to oxidative stress, mitochondrial dysfunction, and increased DNA damage [15,20,27,30,33]. Across the included studies, these effects were observed in a wide range of nanoplatforms and experimental models and were frequently accompanied by markers of persistent DNA damage and impaired cellular recovery after irradiation [15,20,27,30,33,34].

#### 3.5.2. DNA Damage and DNA Repair Inhibition

Enhanced DNA damage, particularly in the form of double-strand breaks, was frequently reported following nanoparticle-assisted irradiation [15,20,27,52]. Increased γ-H2AX foci formation and other markers of DNA damage were commonly used to assess this effect. Beyond this effect, several nanoplatforms were specifically designed to inhibit DNA repair pathways, thereby prolonging damage persistence and increasing radiosensitivity [15,52,74].

Several studies also reported modulation of DNA repair pathways, including effects on components of the DNA damage response such as *RAD50* [52,74]. These findings were observed alongside enhanced radiation responses across multiple experimental systems.

#### 3.5.3. Tumor Microenvironment Modulation

Modulation of the tumor microenvironment was reported in a subset of studies. Hypoxia-targeting approaches, particularly MnO_2_-based systems, were described to increase oxygen availability within tumor models [17,21,53].

Additional strategies included depletion of intracellular glutathione (GSH) and modulation of redox balance [17,19,45,47,68], as well as metabolic interventions targeting glycolysis or cellular energetics [49,69,75,76].

#### 3.5.4. Regulated Cell Death Pathways

Activation of cell death pathways was frequently reported following nanoparticle-assisted radiotherapy. Apoptosis was the most commonly described form of cell death [20,27,30,33].

Ferroptosis was reported in several studies, particularly in association with redox modulation and glutathione depletion [15,17,68,77,78], while additional pathways such as pyroptosis were also described [16,39]. Several studies reported the involvement of multiple cell death pathways within the same experimental system [17,39,68].

#### 3.5.5. Immune Activation and Systemic Effects

Immune-related effects were reported in a subset of studies. Activation of immunogenic cell death markers and cGAS–STING-associated signaling pathways was described following nanoparticle-assisted irradiation [17,21,23,36].

Some studies also reported systemic effects, including responses in non-irradiated tumor sites [17,22,36]. Immune-modulating strategies, including checkpoint-related approaches, were described in combination with radiotherapy in selected nanoplatforms [17,21,23,26,39,56]. The principal associated with nanoparticle-enhanced radiation responses reported across all included studies are summarized in Table 2.

### 3.6. Therapeutic Outcomes

#### 3.6.1. In Vitro Radiosensitization Outcomes

In vitro studies frequently reported enhanced radiation responses across a wide range of nanoparticle platforms. Clonogenic survival assays demonstrated reduced survival fractions in nanoparticle-treated cells compared to radiotherapy alone [20,30,33,79].

Additional findings included decreased cell viability and increased apoptosis following combined nanoparticle and radiotherapy treatment [20,27,33,50,59,72]. Enhanced DNA damage was further supported by increased γ-H2AX foci formation and related markers [15,20,52,74].

Several studies reported radiosensitization metrics such as dose enhancement factors or sensitizer enhancement ratios (SER), indicating increased radiation response in the presence of nanoparticles [24,27,64].

#### 3.6.2. In Vivo Antitumor Efficacy

In vivo studies reported enhanced antitumor effects with nanoparticle-assisted radiotherapy across multiple experimental models. Combined treatment was associated with greater tumor growth delay or regression compared to radiotherapy alone [17,20,39,40,43,50,63,80].

In some studies, marked tumor regression or complete tumor responses were described in a subset of treated animals [13,17,62,67]. Enhanced treatment effects at lower radiation doses were also reported in selected studies [24,27,81,82].

Safety assessments were reported in a proportion of studies, with limited systemic toxicity observed based on body weight, organ histology, and hematological parameters [34,40,48,83].

#### 3.6.3. Survival and Systemic Effects

Several studies reported survival outcomes following nanoparticle-assisted radiotherapy. Prolonged survival or improved overall survival was described in combined treatment groups compared to controls [17,36,43,53,80].

Inhibition of metastatic progression and systemic tumor control were also reported in selected studies [22,43,45]. These findings were described alongside immune-related effects, including responses in non-irradiated tumor sites [17,22,36]. The therapeutic outcomes associated with nanoparticle-assisted radiotherapy across all included studies are summarized in Table 3.

Representative studies provide illustrative examples of the therapeutic outcomes reported for predominantly physical high-Z and multifunctional nanoplatforms. Among relatively simple high-Z systems, Swanner et al. demonstrated that silver nanoparticles significantly enhanced clonogenic radiosensitivity and improved in vivo tumor growth inhibition following irradiation [30]. Bhattarai et al. reported that CXCR4-targeted gold nanoparticles achieved a dose enhancement factor (DEF10) of 1.20 in both MDA-MB-231 and HTB-123 cells and produced sustained tumor regression without regrowth during 50 days of follow-up [20]. Atkinson et al. further showed that gold nanoparticles enhanced radiation response primarily by impairing DNA double-strand break repair rather than increasing the initial number of DNA double-strand breaks [74]. In contrast, representative multifunctional nanoplatforms frequently demonstrated broader therapeutic outcome profiles. Au–Pt bimetallic nanoparticles reduced 4T1 tumor volume from 459.5 ± 76.09 mm^3^ after irradiation alone to 231.7 ± 63.35 mm^3^ following combined treatment without evident systemic toxicity [84]. HfO_2_@MnO_2_–GOx nanoplatforms markedly reduced clonogenic survival, inhibited primary tumor growth by approximately 67%, and suppressed distant tumor growth by up to 98.7% [17], whereas Au@AgBiS_2_-PEG nanoparticles promoted immune activation, reduced pulmonary metastases, and prolonged survival, with approximately 50% of treated animals alive at 60 days compared with complete mortality in the control groups within 14–43 days [39]. These representative examples illustrate the broader range of biological and therapeutic effects reported for multifunctional nanoplatforms; however, direct comparison between platform classes was not feasible because of substantial heterogeneity in nanoparticle design, irradiation protocols, experimental models, and outcome measures.

### 3.7. Translational and Methodological Considerations

Substantial heterogeneity was observed across studies in nanoparticle composition, functionalization strategies, experimental models, irradiation protocols, and outcome assessment methods, limiting direct cross-study comparisons. Radiotherapy protocols were frequently incompletely reported, particularly regarding dose rate, fractionation, beam energy, and treatment timing. In addition, validated radiobiological endpoints such as clonogenic survival assays, sensitizer enhancement ratio (SER), and dose enhancement factor (DEF) were inconsistently reported, limiting the ability to distinguish formal radiosensitization from broader radiation-associated therapeutic enhancement across studies.

Internal validity safeguards, including randomization, blinding, and sample size justification, were infrequently described. Although a minority of studies incorporated advanced experimental designs, including orthotopic, metastatic, dual-tumor, or immunocompetent models, most investigations relied on conventional in vitro platforms and simplified subcutaneous tumor models.

The increasing structural complexity of integrated nanoplatforms was also noted, with implications for reproducibility, scalability, and translational development. Collectively, the included studies contributed to the mechanistic, methodological, and therapeutic patterns summarized in Table 1, Table 2 and Table 3 and Supplementary Tables S3–S5.

## 4. Discussion

### 4.1. Overview of Radiosensitization Mechanisms

This systematic review of 66 preclinical studies demonstrates substantial heterogeneity in nanoplatform design, functionalization strategies, experimental models, and radiotherapy protocols. Across studies, enhanced radiation responses was most frequently associated with high-atomic-number (high-Z)–mediated energy deposition, increased reactive oxygen species (ROS) generation, and enhanced DNA damage following irradiation, while additional mechanisms, including redox modulation, hypoxia targeting, regulated cell death pathways, and immune-related effects, were described in a subset of studies. Collectively, these findings indicate that nanoparticle-mediated enhancement of radiation response results from the interaction of multiple physicochemical and biological processes rather than from a single dominant mechanism.

These observations are consistent with established radiobiological principles in which ionizing radiation induces direct DNA damage and indirect oxidative injury through ROS generated by water radiolysis [4,9,85]. High-Z nanomaterials amplify these effects by increasing local energy deposition and secondary electron generation, thereby enhancing intracellular ROS production and DNA damage [10,85,86,88]. Experimental studies further support these mechanisms, with gold nanoparticles demonstrating dose enhancement factors of approximately 1.3 under kilovoltage irradiation in breast cancer models, while more advanced multifunctional systems achieve sensitizer enhancement ratios exceeding 1.5 through combined oxidative and intracellular damage pathways [14,23,87,89]. In addition to physical dose amplification, several contemporary nanoplatforms incorporate catalytic or chemodynamic mechanisms capable of sustaining oxidative stress through endogenous hydrogen peroxide conversion and glutathione depletion [31,87,90]. Continuous ROS generation, rather than transient oxidative bursts, may represent a more effective paradigm for nanoparticle-mediated radiotherapy enhancement. In catalytic and chemodynamic systems, sustained intracellular ROS production can maintain oxidative stress beyond the initial irradiation event, promoting persistent DNA damage, delaying repair processes, and increasing the probability of irreversible reproductive cell death [17,31,68,77,87,90]. This effect may be further enhanced by stimulus-responsive nanoplatforms capable of depleting intracellular antioxidants such as glutathione or exploiting endogenous hydrogen peroxide to continuously generate reactive species [17,19,21,31,53,68,87,90]. Consequently, the therapeutic benefit of these systems may derive not only from amplification of the initial radiation-induced oxidative insult, but also from prolonged disruption of redox homeostasis that reinforces radiation-associated cellular damage over time [17,21,31,68,77,87,90]. These observations support the emerging view that durable disruption of tumor redox homeostasis may be more therapeutically relevant than transient ROS amplification alone, particularly in tumors with high antioxidant capacity and adaptive stress-response pathways [31,68,77,87,90].

Immune-related effects, including cGAS–STING activation and increased immune cell infiltration, have also been described following nanoparticle-assisted irradiation [18,23,26,91]. Beyond direct radiosensitization, emerging evidence suggests that nanoparticle-assisted radiotherapy may amplify radiation-induced antitumor immunity. By enhancing radiation-induced oxidative stress and DNA damage, nanoplatforms can increase the induction of immunogenic cell death (ICD), characterized by the release of damage-associated molecular patterns (DAMPs), including calreticulin exposure, ATP secretion, and HMGB1 release. These signals promote dendritic-cell recruitment, maturation, and antigen uptake, facilitating cross-presentation of tumor-associated antigens and activation of cytotoxic T-cell responses. Simultaneously, increased DNA damage may result in accumulation of cytosolic double-stranded DNA fragments, leading to activation of the cGAS–STING pathway and subsequent production of type I interferons, which further support dendritic-cell activation and T-cell priming. Through these interconnected mechanisms, nanoparticle-assisted radiotherapy may convert localized tumor irradiation into a systemic immune stimulus capable of enhancing antitumor immunity beyond the irradiated field. Although definitive abscopal responses remain uncommon in breast cancer models, increasing evidence for ICD induction, cGAS–STING activation, and immune-cell infiltration suggests that immune modulation represents an important component of nanoparticle-mediated radiosensitization [92]. Together, these findings support a conceptual shift in the field from purely physical radiosensitization toward integrated biological and microenvironmental modulation.

**Figure 3:**
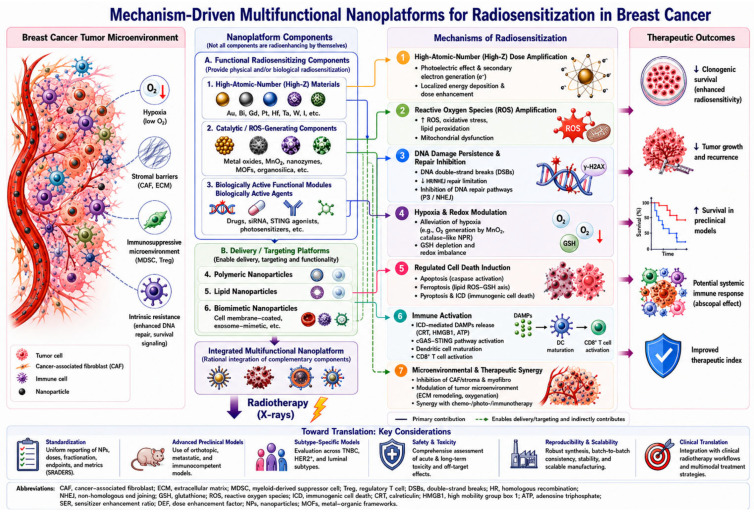
Mechanism-driven multifunctional nanoplatforms for radiosensitization in breast cancer. This figure illustrates how nanoparticle-assisted radiotherapy enhances tumor response through the rational integration of complementary physical, chemical, biological, and immunological mechanisms. Functional radiosensitizing components, including high-atomic-number materials, catalytic/ROS-generating components, and biologically active agents, provide physical dose amplification and/or biological modulation of radiosensitivity. In contrast, delivery and targeting platforms, including polymeric, lipid, and biomimetic nanoparticles, do not necessarily act as radiosensitizers by themselves but facilitate tumor delivery, cellular uptake, targeting, and integration of functional components. Integrated multifunctional nanoplatforms may enhance radiotherapy through high-Z dose amplification, ROS generation, persistent DNA damage, hypoxia and redox modulation, regulated cell death, immune activation, and microenvironmental remodeling. These coordinated mechanisms can lead to reduced clonogenic survival, tumor growth inhibition, improved survival in preclinical models, potential systemic antitumor effects, and an improved therapeutic index. Upward and downward arrows indicate an increase and decrease, respectively, in the level, activity, or biological effect of the corresponding molecule or outcome. Image generated via Canva Pty Ltd., Sydney, Australia (https://www.canva.com/magic-design/, accessed on 7 July 2026).

From a mechanistic perspective, the available evidence suggests that the most effective nanoparticle-assisted radiation enhancement strategies arise from the coordinated integration of complementary mechanisms. While ROS amplification and DNA damage remain the most consistently reported foundational processes [15,20,30,33,63], nanoplatforms combining physical dose enhancement with biologically active components, such as hypoxia-targeting systems, catalytic redox modulators, or immune-related approaches, demonstrate more robust and sustained antitumor effects across preclinical models [17,19,21,26,39,45,93]. These observations indicate that therapeutic enhancement following nanoparticle-assisted irradiation is driven not by nanoparticle composition alone, but by the coordinated engagement of physical, metabolic, and microenvironmental processes that reinforce radiation-induced damage. The relative contribution and integration of these mechanisms across nanoplatforms are summarized in Figure 3.

### 4.2. Functional Categories of Nanoparticle-Mediated Radiosensitization

Beyond classification according to material composition, nanoparticle-mediated radiosensitization can be functionally categorized according to the dominant mechanism through which nanoplatforms interact with ionizing radiation. Across the included studies, four major functional paradigms of nanoparticle-mediated radiotherapy enhancement were identified: conventional radiotherapy enhancement, radiodynamic therapy (RDT), radio-chemodynamic therapy (R-CDT), and multifunctional integrated radiosensitization systems.

#### 4.2.1. Conventional Radiotherapy Enhancement

The earliest and most extensively investigated approach is conventional radiotherapy enhancement based on high-atomic-number (high-Z) nanomaterials, including gold, silver, platinum, bismuth, gadolinium, and hafnium-containing nanoparticles [15,20,27,28,30,33,34,36,50,63]. These systems primarily function through physical interactions with ionizing radiation. Owing to their high electron density, they increase local X-ray absorption and promote the generation of secondary electrons, including photoelectrons and Auger electrons, resulting in amplified energy deposition within irradiated tumor tissues [10,84,85,86].

At the cellular level, enhanced secondary electron generation increases ROS production and radiation-induced DNA damage, particularly double-strand breaks, leading to reduced clonogenic survival and increased apoptosis [15,20,27,30,33]. In studies reporting formal radiobiological endpoints, these systems demonstrated measurable radiosensitization, with reported dose enhancement factors generally ranging between 1.2 and 1.4 under experimental conditions [14,89]. However, despite their effectiveness in amplifying radiation-induced injury, purely physical radiosensitizers often demonstrate limited ability to overcome major determinants of radioresistance, including tumor hypoxia, redox buffering capacity, and microenvironment-mediated protection [4,85,93]. Consequently, although conventional radioenhancement represents the foundation of nanoparticle-mediated radiosensitization, it rarely provides the most pronounced therapeutic responses observed in contemporary studies.

#### 4.2.2. Radiodynamic Therapy

Radiodynamic therapy (RDT) represents an evolution beyond simple physical dose amplification. In RDT systems, X-ray irradiation activates radiosensitizing or photosensitizer-like components incorporated within nanoplatforms, resulting in active ROS generation through radiation-triggered photochemical processes [39,40,41,68]. Metal–organic frameworks (MOFs), porphyrin-containing nanostructures, and several hybrid nanoplatforms exemplify this strategy [39,40,41]. Recent studies have further extended MOF-based radiosensitizers toward multifunctional and stimulus-responsive systems. For example, Zhen et al. developed a mixed-ligand Hf-based nanoscale MOF (Hf-DBP-QP-SN) that combined radiotherapy enhancement, radiodynamic therapy, and X-ray-triggered release of the chemotherapeutic SN38. In a 4T1 breast cancer model, this platform achieved 95.2% tumor growth suppression under low-dose irradiation without evidence of systemic toxicity, illustrating the potential of ionizing radiation-mediated chemistry for on-demand activation of nanotherapeutics and synergistic radiotherapy–chemotherapy combinations [94].

Unlike conventional high-Z radiosensitizers, in which ROS generation occurs primarily as a consequence of radiation absorption, RDT platforms are specifically designed to convert radiation energy into amplified oxidative stress. This strategy promotes increased singlet oxygen production, mitochondrial dysfunction, oxidative injury, and apoptosis [39,40,68]. Collectively, these findings demonstrate that RDT extends radiation-associated biological enhancement beyond physical dose amplification by actively amplifying oxidative damage within irradiated tumors. Importantly, several studies also reported formal radiosensitization using established radiobiological endpoints, supporting the therapeutic potential of this strategy.

#### 4.2.3. Radio-Chemodynamic Therapy

A third functional category comprises radio-chemodynamic therapy (R-CDT) systems, which combine irradiation with catalytic ROS-generating reactions [17,19,21,31,53,87,90]. These platforms typically incorporate catalytic components, including manganese-, iron-, or copper-containing materials, that exploit endogenous hydrogen peroxide within the tumor microenvironment to generate highly reactive hydroxyl radicals through Fenton or Fenton-like reactions [87,90].

At the chemistry level, these systems transform the tumor microenvironment into a continuous source of oxidative stress. In addition to generating ROS, many catalytic platforms simultaneously deplete intracellular glutathione, thereby disrupting antioxidant defenses and enhancing susceptibility to radiation-induced injury [17,31,87,90]. This dual mechanism extends oxidative damage beyond the irradiation period and creates sustained redox imbalance within tumor cells. Several included studies reported enhanced DNA damage, reduced clonogenic survival, increased apoptosis, and superior tumor growth inhibition compared with conventional high-Z systems [17,19,21,53]. These findings suggest that catalytic amplification of oxidative stress represents one of the most effective approaches for overcoming biological radioresistance and enhancing radiation response, although formal radiosensitization was not uniformly demonstrated across studies.

#### 4.2.4. Multifunctional Integrated Radiosensitization Systems

The most advanced category comprises multifunctional nanoplatforms integrating multiple complementary radiosensitization strategies within a single system [17,21,23,26,39,40,45,52,53]. These platforms combine physical dose enhancement with one or more biologically active mechanisms, including catalytic ROS generation, hypoxia modulation, DNA repair inhibition, ferroptosis induction, drug delivery, or immune activation.

From a design perspective, the development of multifunctional systems reflects recognition that tumor radioresistance is multifactorial and cannot be effectively addressed through a single intervention [4,6,93]. Accordingly, many contemporary nanoplatforms simultaneously target several resistance pathways. Examples include oxygen-generating catalytic nanoparticles that alleviate hypoxia while enhancing ROS production [17,19,21], drug-loaded systems that combine radiotherapy with chemotherapy or DNA repair inhibition [23,45,52], and immune-active nanoplatforms capable of promoting dendritic cell maturation, cGAS–STING activation, and CD8^+^ T-cell infiltration following irradiation [23,26,91].

Importantly, the strongest therapeutic outcomes reported in this review were generally associated with these integrated systems. In addition to enhanced radiotherapeutic efficacy, several studies demonstrated durable tumor growth inhibition, suppression of metastatic progression, prolonged survival, and evidence of systemic antitumor immunity [23,26,39,45]. These observations indicate that the most effective nanoparticle-assisted radiotherapy strategies arise not from the dominance of a single mechanism but from the coordinated interaction of complementary physical, biological, and microenvironmental processes.

The emergence of hybrid and multifunctional nanoplatforms reflects the limitations of conventional high-atomic-number (high-Z) radioenhancement as a standalone strategy. Although high-Z materials effectively increase local radiation absorption, secondary electron generation, and ROS production, they do not directly address major biological determinants of radioresistance, including tumor hypoxia, antioxidant defenses, DNA repair capacity, immune suppression, and heterogeneous nanoparticle delivery [4,12,85,93]. Hybrid systems were therefore developed to combine the favorable physical properties of high-Z materials with additional functionalities capable of modulating the tumor microenvironment, enhancing tumor targeting, or amplifying oxidative stress through catalytic mechanisms [17,19,21,39,40]. Multifunctional nanoplatforms represent a further stage of design evolution, integrating several complementary strategies such as radiodynamic therapy, chemodynamic therapy, controlled drug delivery, hypoxia alleviation, ferroptosis induction, DNA repair inhibition, and immune activation within a single construct [17,21,23,26,39,45,52,53]. Across the included studies, the most pronounced therapeutic responses, including greater tumor growth inhibition, improved survival, suppression of metastatic progression, and induction of systemic antitumor immunity, were generally associated with such integrated systems. Taken together, these functional categories illustrate distinct therapeutic profiles across the principal classes of nanoplatforms, although direct quantitative comparison remains limited by methodological heterogeneity. Direct quantitative comparison between major nanomaterial classes was not methodologically feasible because formal radiosensitization metrics (SER or DEF) were reported in only 5 of the 66 included studies, whereas most investigations used heterogeneous endpoints and substantially different experimental protocols. Consequently, the relative therapeutic characteristics of high-Z metals, lipid/polymer systems, MOF-based platforms, and hybrid or multifunctional nanoplatforms can only be interpreted qualitatively rather than through calculation of category-level average radiosensitization effects. The comparisons presented here therefore represent a qualitative synthesis of the available evidence rather than conclusions derived from direct quantitative analyses.

Importantly, these observations suggest that the superiority of multifunctional nanoplatforms derives not from increasing structural complexity per se, but from their ability to simultaneously target multiple interconnected mechanisms of radiation resistance. Future development should therefore prioritize rational mechanistic integration and clinical feasibility rather than the addition of increasingly complex components without a clear biological purpose.

### 4.3. Evolution of Nanoplatform Design

Building on the functional radiosensitization strategies discussed above, the evolution of nanoparticle-mediated radiosensitization in breast cancer reflects a progressive shift from physically driven radioenhancement toward biologically informed platform design. Early studies predominantly focused on high-atomic-number (high-Z) materials, including gold, silver, gadolinium, and hafnium-based nanoparticles, with the primary objective of maximizing radiation energy deposition and secondary electron generation [9,85,95,96]. These systems established the proof-of-concept that nanomaterials could enhance radiation-induced damage, but they also highlighted important limitations related to tumor hypoxia, antioxidant defenses, heterogeneous intratumoral distribution, and variable nanoparticle accumulation [11,12,25,95,97].

As understanding of the biological determinants of radioresistance improved, nanoparticle design increasingly incorporated functional features intended to address these limitations. Surface modifications, including polyethylene glycol coatings, targeting ligands, and biomimetic carriers, were introduced to improve circulation time, tumor uptake, and cellular internalization [98,99,100]. Subsequently, catalytic and microenvironment-responsive systems emerged to modulate tumor hypoxia, redox balance, and oxidative stress dynamics, thereby extending radiosensitization beyond simple physical dose amplification [17,19,21,31,53].

An important development in the evolution of nanoplatform engineering has been the emergence of bimetallic nanoplatforms that combine complementary physicochemical and biological properties within a single construct. These platforms extend the concept of conventional high-Z radioenhancers by integrating physical dose amplification with biologically active functions capable of modulating the tumor microenvironment. Representative examples include Au–Pt bimetallic nanoparticles, which combine the high X-ray absorption properties of gold with platinum-mediated catalytic activity, thereby promoting oxygen generation through decomposition of endogenous hydrogen peroxide while enhancing radiation-induced DNA damage [85]. Similarly, the Fe_3_O_4_–Au bimetallic system included in the present review [80] combined magnetic iron oxide and gold nanoparticles as a multifunctional platform for targeted chemoradiotherapy in 4T1 breast cancer models. More recently, RuCu bimetallic nanozymes demonstrated dual peroxidase-like and catalase-like activities, simultaneously enhancing ROS production and alleviating tumor hypoxia under irradiation in MDA-MB-231 breast cancer models [101]. Collectively, these examples illustrate how bimetallic nanoplatforms bridge the transition from first-generation high-Z radioenhancers toward multifunctional systems integrating complementary physicochemical and biological mechanisms. Rather than relying exclusively on increased local energy deposition, these platforms increasingly exploit coordinated modulation of oxidative stress, tumor oxygenation, and intracellular redox homeostasis to enhance the biological response to irradiation.

More recently, nanoplatform development has increasingly focused on integrating multiple complementary functions within a single construct. Contemporary systems frequently combine physical radioenhancement with catalytic ROS generation, controlled drug delivery, ferroptosis induction, DNA repair inhibition, or immune modulation [17,21,23,26,39,45,52,53]. This evolution reflects a broader conceptual transition in the field: rather than maximizing radiation absorption alone, current strategies seek to overcome multiple interconnected determinants of radioresistance simultaneously.

Comparison across the major nanoplatform classes suggests that no single material category consistently outperformed all others. Rather, therapeutic efficacy appeared to correlate more strongly with the degree of mechanistic integration than with material composition alone. Conventional high-Z nanoplatforms generally demonstrated robust physical radioenhancement but more limited biological activity, whereas lipid- and polymer-based systems primarily contributed through improved delivery, targeting, and pharmacokinetic optimization. In contrast, representative hybrid, multifunctional, and selected bimetallic nanoplatforms integrating high-Z dose amplification with catalytic, microenvironment-responsive, immune-related, or drug-delivery mechanisms more frequently demonstrated broader therapeutic outcome profiles, including enhanced tumor growth inhibition, suppression of metastatic disease, prolonged survival, and immune activation [16,17,21,26,39,40,45,52,53]. Although direct comparison between platform classes was not feasible because of substantial heterogeneity in nanoparticle design, irradiation protocols, experimental models, and outcome assessment, these observations are consistent with the progressive evolution of nanoparticle-mediated radiosensitization toward integrated physicobiological strategies rather than physical dose amplification alone.

Collectively, these observations suggest that the evolution of nanoparticle-mediated radiosensitization has been driven less by changes in material composition alone than by the progressive integration of complementary physicochemical and biological mechanisms capable of overcoming multiple determinants of tumor radioresistance. Importantly, this should not be interpreted as an argument for unlimited nanoparticle complexity. Instead, the available evidence indicates that successful platform design depends on the rational integration of biologically relevant functions that address specific resistance mechanisms. Future development will likely benefit from balancing mechanistic sophistication with manufacturability, reproducibility, safety, and clinical feasibility, thereby enabling the translation of effective radiosensitization strategies into clinically deployable nanomedicines.

### 4.4. Mechanistic Convergence in Radiosensitization

A central finding of this review is the convergence of diverse nanoplatforms toward a limited set of interconnected biological processes associated with enhanced radiation responses, particularly ROS amplification, DNA damage persistence, redox imbalance, and immune-related effects. High-Z nanoparticles enhance ROS generation by increasing local energy deposition and secondary electron emission, thereby amplifying free radical formation and oxidative stress following irradiation [9,10,86,88,102,103]. Several studies further describe catalytic or redox-responsive systems capable of sustaining oxidative injury through glutathione depletion and endogenous hydroxyl radical generation [31,87,90]. These effects are consistently associated with increased DNA damage, impaired repair capacity, reduced clonogenic survival, and enhanced tumor cell death [23,52].

Beyond apoptosis, several studies also reported activation of ferroptosis- and pyroptosis-related pathways, particularly in association with glutathione depletion, lipid peroxidation, and oxidative stress amplification [15,16,17,39,68,77]. Immune-related effects, including immunogenic cell death, dendritic cell activation, macrophage polarization, and cGAS–STING signaling, were also described in selected studies [23,26,91]. Importantly, the most pronounced therapeutic responses were generally observed in multifunctional systems capable of engaging multiple mechanisms simultaneously, particularly combinations of oxidative stress amplification, microenvironment modulation, and immune activation [17,21,26,39,45,52].

Interpretation of these mechanisms should be approached with caution because mechanistic validation was inconsistent across studies. In many cases, pathway involvement was inferred from associative biomarkers rather than confirmed through pathway-specific inhibition or rescue experiments. Nevertheless, the overall evidence supports the concept that effective nanoparticle-mediated radiosensitization in breast cancer emerges from coordinated integration of physical dose amplification, redox modulation, DNA damage persistence, and tumor microenvironment targeting rather than from single-mechanism approaches alone.

### 4.5. Subtype Representation and Biological Context

A notable feature of the current evidence base is the predominance of triple-negative breast cancer (TNBC) models, particularly MDA-MB-231 and murine 4T1 systems, whereas HER2-positive and luminal subtypes remain comparatively underrepresented. Direct subtype-comparative experimental designs were uncommon.

This distribution reflects the aggressive biology of TNBC, which is characterized by genomic instability, hypoxia, dysregulated DNA repair, oxidative stress adaptation, and an immunosuppressive tumor microenvironment, all of which contribute to radioresistance and provide a strong rationale for nanoparticle-assisted strategies designed to enhance radiation response and radiosensitization [2,5,7,93]. Consistent with these vulnerabilities, many of the more advanced nanoplatforms identified in this review—including systems targeting sustained ROS amplification, redox disruption, ferroptosis-associated pathways, and immune activation—were preferentially evaluated in TNBC models [23,26,90].

However, the predominance of TNBC systems limits broader applicability across the molecular spectrum of breast cancer, particularly given the relative underrepresentation of hormone receptor-positive and HER2-positive tumors despite their major clinical relevance [2,7,93]. In addition, the frequent use of established cell lines and murine 4T1 models may not fully capture tumor heterogeneity or microenvironmental complexity. Orthotopic, metastatic, and immunocompetent systems were used less frequently, despite their importance for evaluating immune-mediated enhancement of radiation response, systemic antitumor effects, and translational relevance.

Taken together, these findings indicate that model selection substantially influences both the magnitude and interpretation of radiation response enhancement and reported radiosensitization effects. Greater use of subtype-diverse and clinically representative experimental systems will therefore be essential to define the translational potential of nanoparticle-mediated radiosensitization across breast cancer subtypes.

### 4.6. Therapeutic Efficacy and Radiobiological Validation

Across the included studies, nanoparticle-assisted radiotherapy was frequently associated with enhanced therapeutic activity compared with radiotherapy alone, including reduced cell viability, decreased clonogenic survival, increased apoptosis, enhanced DNA damage, delayed tumor growth, and, in selected studies, improved survival outcomes [14,23,26,87,96,104,105]. Quantitative analyses further demonstrated measurable radiosensitization effects, with sensitizer enhancement ratios generally ranging from approximately 1.3 to 1.6 depending on nanoparticle composition and experimental conditions [14,23,89]. Several studies also reported enhanced antitumor effects at lower radiation doses, suggesting the potential for dose reduction while maintaining therapeutic efficacy [23].

The strength of evidence supporting nanoparticle-mediated radiosensitization varied considerably across studies because rigorous radiobiological validation was not uniformly performed. In the present review, clonogenic survival assays were reported in 26 of 66 studies (39.4%), whereas formal radiosensitization metrics, including sensitizer enhancement ratio (SER) and dose enhancement factor (DEF), were reported in only 5 of 66 studies (7.6%). Although these validated radiobiological endpoints were available in a subset of studies, many investigations relied primarily on surrogate outcome measures. This distinction is particularly important when comparing nanoplatform performance, as therapeutic efficacy and mechanistic activity do not necessarily correlate with formally demonstrated radiosensitization.

Therapeutic efficacy also appeared strongly influenced by nanoplatform complexity and functional integration. While many studies demonstrated increased ROS generation and DNA damage following nanoparticle-assisted irradiation, the most consistent antitumor effects were observed with integrated nanoplatforms combining high-atomic-number–mediated dose amplification with catalytic ROS generation, redox modulation, hypoxia targeting, drug delivery, or immune-modulating strategies [17,21,23,39,40,45,52,68,76,77]. These findings suggest that durable therapeutic enhancement following nanoparticle-assisted irradiation depends not on a single functional property, but on coordinated integration of complementary physical and biological mechanisms.

### 4.7. Methodological Limitations of Preclinical Evidence

Despite the substantial growth of preclinical research in nanoparticle-mediated radiosensitization, the available evidence remains limited by considerable methodological heterogeneity. Major differences in nanoparticle composition, particle size, surface functionalization, therapeutic payloads, irradiation protocols, experimental models, and outcome assessment methods [15,20,24,33,37,45,52,68,76,81] substantially complicate direct cross-study comparison and limit quantitative synthesis. Radiation parameters were inconsistently reported, with considerable variability in total dose, fractionation schedules, beam energy, dose rate, and irradiation modality [15,20,24,30,52,63,74], while detailed dosimetric characterization was frequently lacking, reducing reproducibility and limiting radiobiological interpretation. This limitation is particularly relevant for nanoparticle-mediated radiosensitization because the magnitude of physical dose enhancement depends on radiation quality [9,10,85,86]. High-atomic-number (high-Z) nanomaterials interact differently with kilovoltage and megavoltage photon beams, influencing photoelectric absorption, secondary electron generation, and local energy deposition—the principal physical mechanisms underlying nanoparticle-mediated radiotherapy enhancement [9,10,85,86,88]. Consequently, incomplete reporting of beam energy, dose rate, fractionation, and irradiation modality not only reduces reproducibility but also limits meaningful comparison of both the physical and biological effects reported across studies. Greater standardization of radiotherapy protocols and more comprehensive reporting of irradiation parameters will therefore be essential to improve cross-study comparability and facilitate clinical translation.

Another important limitation is the inconsistent use of rigorous radiobiological endpoints. In addition, mechanistic interpretation was frequently based on associative biomarkers such as ROS generation, γ-H2AX expression, oxidative stress markers, or hypoxia-related measurements. While these findings provide valuable mechanistic clues, they do not by themselves establish a causal contribution to radiosensitization. Pathway-specific inhibition studies, rescue experiments, and genetic validation approaches were infrequently performed. Consequently, many reported mechanisms should be interpreted as indicative of pathway involvement rather than definitive mechanistic drivers of radiosensitization.

Although many studies described reduced viability, increased apoptosis, oxidative stress, or enhanced DNA damage following irradiation [17,19,21,31,39,68,77], relatively few incorporated clonogenic survival assays, sensitizer enhancement ratios (SER), dose enhancement factors (DEF), or standardized radiobiological modeling [14,15,20,30,52]. Consequently, formal radiosensitization was often inferred from short-term cytotoxicity, apoptosis, ROS generation, DNA damage markers, or surrogate molecular endpoints rather than validated measures of long-term reproductive cell death such as clonogenic survival assays or quantitative sensitizer enhancement metrics. Mechanistic conclusions also varied in strength, as increased ROS generation, γ-H2AX expression, or hypoxia-related changes were frequently interpreted as evidence of causal pathway involvement without inhibitor-based or rescue experiments confirming mechanistic specificity [17,21,31,39,45,77].

The experimental models used across studies present additional translational limitations. Triple-negative breast cancer models, particularly 4T1 and MDA-MB-231, predominated throughout the literature [15,16,17,20,21,22,23,39,40,45,52,55,56,68,69,74,76,77,81], whereas luminal and HER2-positive subtypes were comparatively underrepresented [24,29,35,51,62]. Although TNBC is highly relevant because of its aggressive biology and relative radioresistance, this imbalance limits the broader applicability of current findings across the molecular spectrum of breast cancer. Moreover, many in vivo studies relied on subcutaneous xenograft or syngeneic murine systems that incompletely reproduce the stromal complexity, immune heterogeneity, and metastatic behavior of human breast cancer.

Several studies additionally employed structurally complex integrated nanoplatforms incorporating multiple coatings, targeting ligands, catalytic components, or combinatorial therapeutic payloads. While these systems often demonstrated strong preclinical efficacy [17,21,23,39,40,45,52,68,76,77], their translational feasibility remains uncertain because biodistribution, pharmacokinetics, long-term toxicity, immunogenicity, manufacturing scalability, and regulatory reproducibility were infrequently evaluated [10,11,13,16,93,94,95,96,97,98,99,100,102]. Toxicity assessment was also inconsistently reported, with relatively few studies providing systematic evaluation of long-term systemic, immunological, or organ-specific adverse effects. Publication bias toward positive radiation-enhancement and radiosensitization findings may further contribute to overestimation of therapeutic benefit.

Taken together, these limitations highlight the need for greater methodological standardization in preclinical nanoparticle-assisted radiotherapy research, including harmonized irradiation protocols, standardized radiobiological endpoints, improved mechanistic validation, and broader use of clinically representative breast cancer models.

### 4.8. Barriers to Clinical Translation

Despite substantial advances in nanoparticle-mediated radiosensitization in preclinical models, clinical translation remains limited. A major barrier is the structural complexity of many nanoplatforms, which frequently incorporate multiple targeting ligands, catalytic elements, and therapeutic payloads. Although these combinatorial systems often demonstrate enhanced efficacy experimentally, their clinical development is challenged by issues related to reproducibility, manufacturing consistency, regulatory approval, scalability, and long-term safety [25,96,97]. Variability in nanoparticle composition, stability, and biodistribution further complicates standardization and regulatory evaluation.

Delivery and tumor accumulation represent additional challenges. Many preclinical studies rely on passive targeting through the enhanced permeability and retention (EPR) effect, which is highly variable and generally less pronounced in human tumors than in experimental models [5,97]. Emerging biologically derived delivery systems may help overcome several of the delivery-related barriers limiting clinical translation of nucleic acid-based radiosensitization strategies. In particular, exosome-based and exosome-mimicking systems have attracted increasing interest because of their intrinsic biocompatibility, tumor-homing potential, and ability to facilitate intracellular transport of functional cargo [106,107,108]. In breast cancer models, exosome-inspired nanoplatforms have been shown to improve tumor localization and enhance radiosensitization through redox modulation and ROS amplification, while engineered HER2-targeting exosomes loaded with *miR-34a* have demonstrated improved tumor selectivity and antitumor activity. Together with advances in nanoparticle-mediated siRNA delivery, these findings suggest that biologically inspired delivery systems may further enhance the therapeutic potential of nucleic acid-based radiosensitization strategies by improving intracellular delivery, protecting RNA cargo from degradation, and reducing off-target effects [109,110,111]. Moreover, differences in vascularization, interstitial pressure, and microenvironmental architecture between animal models and patients may substantially influence nanoparticle distribution and therapeutic efficacy. The route of administration also remains a practical limitation, as intratumoral delivery may not be feasible for all anatomical sites, whereas systemic administration raises concerns regarding off-target accumulation and toxicity [25,97].

Another important challenge is the integration of nanoparticle-assisted radiation-enhancement strategies into established clinical radiotherapy workflows. Differences in radiation energy, fractionation schedules, and treatment planning between preclinical studies and clinical practice complicate direct translation. Additional complexity arises from compatibility with multimodal breast cancer treatment strategies, including chemotherapy, endocrine therapy, HER2-targeted therapy, and immunotherapy, all of which may influence nanoparticle pharmacokinetics, toxicity, and therapeutic sequencing. Furthermore, the absence of standardized protocols for combining nanoplatforms with radiotherapy limits reproducibility and hinders definition of optimal treatment parameters.

Despite these barriers, early clinical translation has been achieved for selected nanoplatforms. Hafnium oxide nanoparticles (NBTXR3/Hensify) represent the most advanced example, demonstrating clinical efficacy in combination with radiotherapy and obtaining regulatory approval for locally advanced soft tissue sarcoma, with ongoing evaluation in additional tumor types [112,113,114,115]. Similarly, gadolinium-based nanoparticles such as AGuIX have progressed into clinical trials, particularly in brain metastases and other indications, demonstrating feasibility, imaging capability, and preliminary therapeutic benefit [114,115,116,117]. However, clinical translation of nanoparticle radiosensitizers in breast cancer remains considerably less advanced than in soft tissue sarcoma or neuro-oncologic applications. One important reason is that NBTXR3 has been successfully developed for tumors amenable to direct intratumoral administration, whereas breast cancer is frequently treated following surgical resection or within multimodal treatment pathways that include systemic therapy, limiting opportunities for local nanoparticle delivery and complicating evaluation of biodistribution within residual microscopic disease [112,113,114,115]. Moreover, breast cancer exhibits marked biological heterogeneity across molecular subtypes, further increasing the complexity of patient selection and treatment optimization [2,7,8]. Although NBTXR3 is currently being evaluated in a phase I dose-escalation/dose-expansion study that includes metastatic triple-negative breast cancer among the expansion cohorts, dedicated breast cancer clinical data remain limited [118,119]. Consequently, successful clinical translation will require reproducible biodistribution, favorable safety profiles, compatibility with contemporary breast cancer treatment pathways, and demonstration of clear advantages over existing radiotherapy strategies [10,11,12,97].

Taken together, these findings suggest that the major obstacle to clinical implementation is not the absence of therapeutic activity following nanoparticle-assisted irradiation, but the gap between experimental complexity and clinical feasibility. The systems most likely to achieve successful translation are not necessarily the most sophisticated platforms, but those combining reproducible manufacturing, predictable biodistribution, acceptable safety, and compatibility with established radiotherapy workflows. Future progress will therefore depend on clinically adaptable nanoplatforms, standardized treatment protocols, and rational selection of disease settings in which localized dose amplification can be safely and reproducibly achieved.

### 4.9. Strengths and Contribution of the Review

This systematic review provides a structured synthesis of 66 preclinical studies investigating nanoparticle-mediated radiosensitization in breast cancer, integrating nanoplatform design features with reported mechanisms and therapeutic outcomes. By linking nanoparticle composition, functionalization strategies, and experimental models with radiobiological and biological endpoints, the review offers a cohesive overview of a highly heterogeneous and rapidly evolving field.

Unlike prior narrative or technology-focused reviews, this analysis directly connects nanoplatform design with functional outcomes, including reactive oxygen species generation, DNA damage persistence, tumor microenvironment modulation, regulated cell death pathways, and immune-related responses. This integrative approach enables cross-study comparison and highlights recurring mechanistic and therapeutic patterns that are not readily apparent in isolated reports.

Importantly, the review also identifies recurrent methodological limitations affecting interpretation of reported radiosensitization and radiation-enhancement effects, including heterogeneity in experimental design, incomplete reporting of radiotherapy parameters, and inconsistent use of validated radiobiological endpoints. By addressing these issues, the present work provides a practical framework for improving methodological rigor, standardization, reproducibility, and translational relevance in future studies.

Collectively, these findings clarify the relationships between nanoparticle design, mechanistic pathways, and therapeutic outcomes, thereby supporting the rational development and evaluation of nanoparticle-based radiosensitizers in breast cancer.

### 4.10. Future Directions and Research Priorities

Future progress in nanoparticle-mediated radiosensitization will depend on the development of simplified integrated nanoplatforms combining physical dose enhancement with biologically and microenvironmentally relevant mechanisms. Key priorities include improved tumor delivery and penetration, clinically applicable targeting strategies, and optimization of pharmacokinetics, biodegradability, and safety to enhance translational feasibility. Equally important is closer alignment between preclinical research and clinical radiotherapy practice through standardized irradiation protocols, validated radiobiological endpoints and radiosensitization metrics, and broader use of orthotopic, metastatic, and immunocompetent models.

A major future priority is to move beyond descriptive mechanistic observations toward rigorous causal validation of the biological processes responsible for enhanced radiation response and formally validated radiosensitization. Although increased ROS generation, DNA damage, ferroptosis-associated markers, and immune activation are frequently reported, their relative contribution to therapeutic efficacy remains incompletely defined. Future studies should therefore incorporate pathway-specific inhibition, genetic knockdown or knockout strategies, rescue experiments, and longitudinal mechanistic analyses to distinguish causal mechanisms from associative biomarkers. Particular attention should be directed toward the interplay among redox regulation, DNA repair pathways, ferroptosis susceptibility, tumor hypoxia, and cGAS–STING-mediated immune activation, as these processes recur across many of the most effective multifunctional nanoplatforms identified in this review.

The logical next stage in the evolution of nanoparticle-assisted radiotherapy is therefore not the addition of further structural complexity but the identification of the minimal combination of complementary functions required to overcome specific mechanisms of radioresistance. Although many contemporary systems incorporate multiple functional components, it remains unclear which elements are essential for therapeutic efficacy and which primarily increase manufacturing complexity and regulatory burden. Mechanism-driven development could identify the minimal combination of functions required to overcome specific determinants of radioresistance within defined biological contexts. Such an approach would support the development of simpler, more reproducible, scalable, and clinically translatable nanoplatforms without compromising therapeutic performance. Accordingly, future radiosensitizer development should prioritize precise mechanistic targeting, manufacturability, safety, and compatibility with contemporary radiotherapy workflows rather than structural complexity alone.

Ultimately, successful clinical translation will require not only technological innovation, but also closer integration of nanoplatform design with tumor biology, pharmacology, and radiotherapy principles. Progress toward standardized, mechanistically validated, radiobiologically rigorous, and clinically aligned research frameworks will be essential to bridge the gap between experimental promise and therapeutic application.

## 5. Conclusions

In summary, the preclinical evidence indicates that nanoparticle-assisted enhancement of radiation response in breast cancer is most effective when physical dose amplification is combined with biologically active mechanisms such as redox modulation, DNA damage persistence, hypoxia targeting, regulated cell death, and immune activation. This integrated strategy appears particularly relevant for aggressive and treatment-resistant subtypes such as TNBC, where hypoxia, genomic instability, and tumor microenvironment-mediated resistance limit the efficacy of conventional radiotherapy. Collectively, the available evidence suggests that the field of nanoparticle-mediated radiosensitization is evolving from predominantly physical radioenhancement toward integrated physicobiological strategies. The therapeutic performance of contemporary nanoplatforms appears to depend less on material composition alone than on the rational integration of complementary mechanisms that simultaneously address multiple determinants of tumor radioresistance. However, further progress will require standardized preclinical validation, rigorous mechanistic confirmation, comprehensive reporting of irradiation protocols, and clinically representative experimental models. Rather than pursuing increasing structural complexity alone, future development should prioritize mechanism-driven, clinically translatable nanoplatforms that balance biological sophistication with manufacturability, reproducibility, safety, and compatibility with modern radiotherapy workflows.

## Figures and Tables

**Table 1:** Characteristics of included studies.

Study	NP Platform	Functionalization/Payload	Experimental Models	Subtype
Sun (2022) [15]	AGuIX NP	Ultrasmall gadolinium-based NP (no additional ligand)	TNBC cells (IV)	TNBC
Liu (2023) [16]	Polymeric NP	Epigenetic drug-loaded system	TNBC (IV/IVV)	TNBC
Hu (2024) [17]	HfO_2_@MnO_2_@GOx NP platform	GOx-doped MnO_2_-coated HfO_2_	4T1 dual-tumor model (IV/IVV)	TNBC
Shao (2024) [19]	Liposomal NP platform	GOx/MnO_2_ co-loaded	Breast cancer (IV/IVV)	TNBC
Bhattarai (2021) [20]	Au NP	CXCR4-targeted peptide functionalization	Breast cancer (IV/IVV)	TNBC
Wang X (2024) [21]	Oxygen-generating NP platform	Catalase-like hypoxia-modulating NP	TNBC (IV/IVV)	TNBC
Chen SF (2025) [22]	CeO_2_ nanobooster	OMV-modified	Metastatic TNBC model (IV/IVV)	TNBC
Samani (2020) [24]	Au nanocluster	Trastuzumab + FA	SK-BR3 (IV)	HER2+
Cui (2017) [27]	Au NP	Cisplatin-combined	TNBC (IV/IVV)	TNBC
Nicol (2018) [28]	Au NP	PEG + RME + H5WYG peptides	MCF-7, MDA-MB-231 (IV/IVV)	Mixed
Abdollahi (2023) [29]	Fe_3_O_4_@Au NP	HER2-targeted magnetic core–shell NP	HER2+ cells (IV)	HER2+
Swanner (2015) [30]	Ag NP	PVP-coated	TNBC models (IV/IVV)	TNBC
Montazersaheb (2024) [31]	Ag NP	Green-synthesized AgNP	TNBC cells (IV)	TNBC
Zhang F (2023) [32]	Pt nanoassembly	Pt(0)/Pt^2+^ coordination nanostructure	Breast cancer (IV/IVV)	General
Rashidzadeh (2023) [33]	Pt NP	Alginate-coated PtNP	Breast tumor model (IV/IVV)	General
Deng (2018) [34]	Bi NP	Folate-inserted RBC membrane coating	4T1 (IV/IVV)	General
Dastgir (2026) [35]	Bi_2_O_3_ NP	Chitosan/5-ALA/CUR or β-CD/glucose	HER2+ models (IV/IVV)	HER2+
Yu (2023) [36]	Gd_2_O_3_ NP	Immune-activating radiosensitizer NP	TNBC (IV/IVV)	TNBC
Nosrati (2023) [37]	Gd_2_O_3_/Au hybrid NP	BSA-capped hybrid nanostructure	Breast cancer models (IV/IVV)	General
Wu (2023) [38]	Fe_3_O_4_-Au hybrid NP	Hsp70-targeting peptide (TPP-PEG4)	TNBC cells (IV)	TNBC
Xiao (2023) [39]	Au@AgBiS_2_ core–shell NP	PEGylated	4T1 (IV/IVV)	TNBC
Wang Y (2025) [40]	Pt@Ce-MOF NP platform	RGD + FA dual-targeting; cisplatin-loaded	4T1 (IV/IVV)	TNBC
Zhang J (2025) [41]	Mesoporous organosilica NP	Gd + DOX-loaded (MOs-G@DOX)	4T1 (IV/IVV)	TNBC
Minafra (2019) [42]	Solid lipid NP	CUR-loaded lipid nanocarrier	Breast cancer cells (IV)	Mixed
Liu TI (2020) [43]	Polymeric nanophototherapeutic NP	SAHA + ICG; ROS-responsive	TNBC (IV/IVV)	TNBC
Chen S (2024) [44]	Telmisartan NP	Erythrocyte membrane-coated	4T1 spheroid + orthotopic (IV/IVV)	TNBC
Yang (2026) [45]	RNAi NP platform	αTrop2; si*MNX1*-AS1; GSH-responsive	TNBC models (IV/IVV)	TNBC
Bromma (2019) [46]	Lipid NP	Gold nanoparticle delivery lipid system	Breast cancer cells (IV)	General
Li P (2026) [47]	Lipid NP	PEGylated sunitinib-loaded (Sun@PL)	4T1 (IVV)	General
Karabuga (2023) [48]	Liposomal radiosensitizer	PEG + FA + QD–Ce6 conjugate	4T1 (IVV)	General
Askar (2021) [49]	MgO NP	HA/FA dual-targeted surface modification	Breast cancer (IV)	General
Zhang Y (2026) [50]	Pt NP	BSA-coated Pt nanoparticle	Breast cancer (IV/IVV)	General
Yamaguchi (2018) [51]	Silica NP	Anti-HER2 antibody-conjugated	SK-BR3 (IV)	HER2+
Zetrini (2024) [52]	siRNA NP	*RAD50*-targeting siRNA delivery system	TNBC models (IV/IVV)	TNBC
Abbasi (2016) [53]	MnO_2_ NP	H_2_O_2_-responsive O_2_ generation	EMT6, MDA-MB-231 (IV/IVV)	Mixed
Nosrati (2022) [54]	Janus Fe_3_O_4_/Bi_2_S_3_ NP	BSA + FA functionalization	4T1 murine model (IV/IVV)	General
Ghaffarlou (2023) [55]	Ag-Ag_2_S Janus NP	BSA + FA	4T1 (IV/IVV)	General
Wang D (2024) [56]	Au/MnO_2_ NP	Cancer-cell membrane + siRNA	4T1 model (IV/IVV)	TNBC
Musielak (2023) [57]	Au NP	Size/shape-dependent functionalization study	MCF-7 (IV)	General
Albers (2025) [58]	BaSO_4_ NP	None	Basal-like mammary model (IVV)	Basal-like
Shiridokht (2025) [59]	Ag NP + chitosan NP	Metformin-loaded chitosan + AgNP	MCF-7 (IV)	General
Hussein (2025) [60]	Chitosan NP	Resveratrol-loaded	MCF-7 + DMBA (IV/IVV)	General
Zhang L (2021) [61]	Au nanocluster	GSH@AuNC + *hNIS* gene delivery	TNBC (IV/IVV)	TNBC
Cline (2021) [62]	Potassium iodide NP	PMAO-coated; NIS-exploiting	Breast tumor models (IV/IVV)	Luminal
Mulgaonkar (2017) [63]	Hollow Au NP	None	TNBC xenograft (IVV)	TNBC
Ghahremani (2018) [64]	Au nanocluster	AS1411 aptamer + BSA coating	4T1 (IV)	General
Kefayat (2019) [65]	Au NP	BSA; FA/glucose/glutamine	4T1 BALB/c (IVV)	General
Detappe (2020) [66]	Ultrasmall gadolinium NP	Anti-MUC1-C antibody (3D1)-conjugated	E0771 model (IVV)	Mixed
Rahmani (2025) [67]	Fe_3_O_4_@ZIF-8 NP	CUR-loaded MOF system	MDA-MB-231 (IV)	TNBC
Shin (2026) [68]	Radio-activatable lipid NP	7-DHC lipid + si*GPX4*	4T1 (IV/IVV)	TNBC
Li M (2021) [69]	Au NP	Glucose-tagged + CUR combination	MDA-MB-231 xenograft (IVV)	TNBC
Kan (2026) [70]	Ag nanocluster	Aptamer-functionalized targeting nanocluster	TNBC (IV/IVV)	TNBC
Zhu (2021) [71]	Iodinated polymersome	SAHA-loaded; redox-sensitive vesicular system	Breast cancer models (IV/IVV)	General
Asadi (2024) [72]	Zinc NP	Alginate-coated; DOX-conjugated	TNBC cells (IV)	TNBC
Mousazadeh (2023) [73]	Ag_2_S NP	Alginate-coated	Breast tumor models (IV/IVV)	General
Atkinson (2025) [74]	Au NP	PEG + transferrin targeting	4T1 (IV)	TNBC
Thabet (2022) [75]	Nanocomposite system	Metabolic pathway-targeting nanoplatform	Breast cancer (IV)	General
Zhang H (2025) [76]	Liposomal NP platform	DSPE-PEG-RGD; GOx + BSO	TNBC models (IV/IVV)	TNBC
Aishajiang (2025) [77]	Hollow Bi_2_Se_3_ nanomedicine	DSPE-PEoz; RSL3 + diABZi	4T1 TNBC (IV/IVV)	TNBC
Shi (2024) [78]	Polymeric nanoadjuvant	BDP-SS-PEG + sorafenib	Breast cancer models (IV/IVV)	TNBC
Mehrnia (2021) [79]	Au NP	AS1411 nucleolin-targeting aptamer	Breast cancer cells (IV)	Mixed
Nosrati (2021) [80]	Fe_3_O_4_-Au heterodimer	BSA + FA + CUR	4T1 (IV/IVV)	TNBC
Nosrati (2022) [81]	Polymeric NP	Gold prodrug nanocarrier system	Breast cancer (IV/IVV)	General
Zhao (2016) [81]	Mesoporous silica-encapsulated Au nanorods	PEGylated + RGD-conjugated targeting	TNBC cells (IV/IVV)	TNBC
Talik (2020) [82]	Bi_2_O_3_ NP	Combined with cisplatin + baicalein fraction	MCF-7, MDA-MB-231 (IV)	Mixed
Colak (2024) [83]	Bi_2_S_3_ NP	Alginate hydrogel-embedded system	Breast tumor model (IVV)	General

Notes: NP = nanoparticle; IV = in vitro; IVV = in vivo; TNBC = triple-negative breast cancer; HER2+ = human epidermal growth factor receptor 2-positive; FA = folic acid; GOx = glucose oxidase; DOX = doxorubicin; CUR = curcumin; PEG = polyethylene glycol; RGD = arginine-glycine-aspartic acid peptide; BSA = bovine serum albumin; PVP = polyvinylpyrrolidone; RBC = red blood cell; OMV = outer membrane vesicle; TPP = targeting peptide; SAHA = suberoylanilide hydroxamic acid; ICG = indocyanine green; RNAi = RNA interference; HA = hyaluronic acid; QD = quantum dot; Ce6 = chlorin e6; PMAO = poly(maleic anhydride-alt-1-octadecene); *hNIS* = human sodium iodide symporter; DMBA = 7,12-dimethylbenz[a]anthracene; MOF = metal–organic framework; siRNA = small interfering RNA; GSH = glutathione; BSO = buthionine sulfoximine; 7-DHC = 7-dehydrocholesterol; PEoz = poly(2-ethyl-2-oxazoline); RSL3 = ferroptosis inducer RSL3; diABZi = STING agonist diamidobenzimidazole; Mixed = multiple breast cancer models/subtypes; General = subtype not specified.

**Table 2:** Mechanisms of Nanoparticle-Mediated Radiosensitization in Breast Cancer.

Study	ROS ↑	DNA ↑	Hypoxia ↓	Ferroptosis/Other RCD	Immune	Targeting	Drug
Sun (2022) [15]	+	±	−	Ferroptosis	−	−	−
Liu (2023) [16]	+	±	−	Pyroptosis	+	−	+
Hu (2024) [17]	+	+	−	Ferroptosis	+	−	+
Shao (2024) [19]	+	+	±	−	−	−	+
Bhattarai (2021) [20]	+	+	−	−	−	+	−
Wang X (2024) [21]	+	±	+	−	−	−	−
Chen (2025) [22]	+	+	±	ICD	+	+	−
Samani (2020) [24]	±	−	−	Apoptosis	−	+	−
Cui (2017) [27]	+	+	−	Apoptosis	−	−	+
Nicol (2018) [28]	+	+	−	−	−	+	−
Abdollahi (2023) [29]	±	±	−	−	−	+	−
Swanner (2015) [30]	+	+	−	Apoptosis	−	−	−
Montazersaheb (2024) [31]	±	±	±	Apoptosis/ER stress	−	−	−
Zhang F (2023) [32]	+	+	−	Apoptosis	−	−	−
Rashidzadeh (2023) [33]	+	±	−	−	−	−	−
Deng (2018) [34]	+	+	−	−	−	+	−
Dastgir (2026) [35]	+	±	−	Apoptosis	−	±	+
Yu (2023) [36]	+	±	−	−	+	−	−
Nosrati (2023) [37]	+	+	−	Apoptosis	−	−	−
Wu (2023) [38]	+	+	−	−	−	+	−
Xiao (2023) [39]	+	+	−	Pyroptosis	+	±	−
Wang Y (2025) [40]	+	+	+	Apoptosis	−	+	+
Zhang J (2025) [41]	+	+	−	Apoptosis	−	−	+
Minafra (2019) [42]	±	−	−	Apoptosis	−	−	+
Liu TI (2020) [43]	+	+	−	Apoptosis/HDAC	±	−	+
Chen (2024) [44]	+	−	+	−	−	+	+
Yang (2026) [45]	+	±	−	−	−	+	+
Bromma (2019) [46]	±	+	−	−	−	−	+
Li P (2026) [47]	−	−	+	−	±	−	+
Karabuga (2023) [48]	+	±	−	PDT-mediated apoptosis	−	+	+
Askar (2021) [49]	±	−	−	−	−	+	−
Zhang Y (2026) [50]	+	±	−	−	−	−	−
Yamaguchi (2018) [51]	±	−	−	−	−	+	−
Zetrini (2024) [52]	−	+	−	−	−	±	+
Abbasi (2016) [53]	+	±	+	−	−	−	−
Nosrati (2022) [54]	+	+	−	Apoptosis	−	+	−
Ghaffarlou (2023) [55]	+	+	−	Apoptosis	−	+	−
Wang D (2024) [56]	+	+	+	±(ICD-related)	+	+	+
Musielak (2023) [57]	+	+	−	−	−	±	−
Albers (2025) [58]	−	−	−	−	−	−	−
Shiridokht (2025) [59]	+	+	−	Apoptosis	−	−	+
Hussein (2025) [60]	±	±	−	Apoptosis	+	−	+
Zhang L (2021) [61]	±	+	−	−	−	+	+
Cline (2021) [62]	±	±	−	−	−	+	+
Mulgaonkar (2017) [63]	+	+	−	−	−	−	−
Ghahremani (2018) [64]	±	−	−	−	−	+	−
Kefayat (2019) [65]	±	−	−	−	−	+	−
Detappe (2020) [66]	±	±	−	−	−	+	−
Rahmani (2025) [67]	+	−	−	Apoptosis	−	−	+
Shin (2026) [68]	+	−	−	Ferroptosis/ICD	+	−	+
Li M (2021) [69]	±	−	±	Apoptosis	−	+	+
Kan (2026) [70]	+	+	−	Apoptosis	−	+	−
Zhu (2021) [71]	+	+	−	Apoptosis	−	−	+
Asadi (2024) [72]	±	−	−	Apoptosis	−	−	+
Mousazadeh (2023) [73]	+	±	−	Apoptosis	−	−	−
Atkinson (2025) [74]	±	+	−	−	−	+	−
Thabet (2022) [75]	±	−	−	−	−	−	−
Zhang H (2025) [76]	+	±	−	−	−	+	+
Aishajiang (2025) [77]	+	+	−	Ferroptosis	+	−	+
Shi (2024) [78]	+	±	−	Ferroptosis	−	−	+
Mehrnia (2021) [79]	±	−	−	−	−	+	−
Nosrati (2021) [80]	+	+	−	−	−	+	+
Nosrati (2022) [81]	+	+	−	Apoptosis	−	−	+
Zhao (2016) [81]	+	+	−	−	−	+	−
Talik (2020) [82]	+	−	−	−	−	−	+
Colak (2024) [83]	+	±	−	−	−	Local	−

Notes: Mechanistic and outcome classifications summarize the findings reported in the original studies. Biological effects such as increased ROS generation, DNA damage, apoptosis, immune activation, or tumor growth inhibition indicate radiation-associated biological enhancement (radioenhancement) but do not, by themselves, establish formal radiosensitization. According to radiobiological principles, formal radiosensitization requires quantitative validation using clonogenic survival assays, sensitizer enhancement ratio (SER), dose enhancement factor (DEF), dose-modifying factor (DMF), or equivalent dose–response analyses. ROS ↑ = increased reactive oxygen species; DNA Damage ↑ = direct DNA double-strand break markers or DNA repair inhibition; Hypoxia ↓ = oxygenation or hypoxia modulation; RCD = regulated cell death (apoptosis, ferroptosis, pyroptosis, ICD, etc.); ICD = immunogenic cell death; “±” = partial, indirect, or secondary evidence; “Local” = passive/localized tumor accumulation (not active targeting).

**Table 3:** Radiobiological and Therapeutic Outcomes Reported in Studies of Nanoparticle-Mediated Radiotherapy in Breast Cancer.

Study	↓ Viability	↑ Apoptosis	↑ DNA Damage	Tumor Growth ↓	Survival ↑	SER/DEF
Sun (2022) [15]	+	±	±	−	−	−
Liu (2023) [16]	+	±	±	+	−	−
Hu (2024) [17]	+	±	+	+	−	−
Shao (2024) [19]	+	±	+	+	−	−
Bhattarai (2021) [20]	+	+	+	+	−	−
Wang X (2024) [21]	+	±	±	+	−	−
Chen (2025) [22]	+	±	+	+	−	−
Samani (2020) [24]	+	+	−	−	−	+
Cui (2017) [27]	+	+	+	+	−	−
Nicol (2018) [28]	+	+	+	+	−	+
Abdollahi (2023) [29]	±	±	±	−	−	−
Swanner (2015) [30]	+	+	+	+	−	−
Montazersaheb (2024) [31]	±	±	±	−	−	−
Zhang F (2023) [32]	+	+	+	+	−	−
Rashidzadeh (2023) [33]	+	±	±	+	−	−
Deng (2018) [34]	+	±	+	+	+	−
Dastgir (2026) [35]	+	+	±	+	−	−
Yu (2023) [36]	+	±	±	+	−	−
Nosrati (2023) [37]	+	+	+	+	−	−
Wu (2023) [38]	+	+	+	−	−	−
Xiao (2023) [39]	+	+	+	+	+	−
Wang Y (2025) [40]	+	+	+	+	−	−
Zhang J (2025) [41]	+	+	+	+	−	−
Minafra (2019) [42]	+	+	−	−	−	−
Liu TI (2020) [43]	+	+	+	+	−	−
Chen (2024) [44]	+	−	−	+	−	−
Yang (2026) [45]	+	±	±	+	−	−
Bromma (2019) [46]	±	−	+	−	−	−
Li P (2026) [47]	−	−	−	+	−	−
Karabuga (2023) [48]	−	±	±	+	−	−
Askar (2021) [49]	±	−	−	−	−	−
Zhang Y (2026) [50]	+	±	±	+	−	−
Yamaguchi (2018) [51]	±	−	−	−	−	−
Zetrini (2024) [52]	+	−	+	+	−	−
Abbasi (2016) [53]	+	±	±	+	−	−
Nosrati (2022) [54]	+	+	+	+	−	−
Ghaffarlou (2023) [55]	+	+	+	+	−	−
Wang D (2024) [56]	+	±	+	+	−	−
Musielak (2023) [57]	±	−	−	−	−	−
Albers (2025) [58]	−	−	−	+	−	−
Shiridokht (2025) [59]	+	+	+	−	−	−
Hussein (2025) [60]	+	+	±	+	−	−
Zhang L (2021) [61]	+	−	+	+	−	−
Cline (2021) [62]	±	−	±	+	−	−
Mulgaonkar (2017) [63]	−	−	±	+	−	−
Ghahremani (2018) [64]	+	−	−	−	−	−
Kefayat (2019) [65]	−	−	−	+	+	−
Detappe (2020) [66]	−	−	±	+	+	−
Rahmani (2025) [67]	+	+	−	−	−	−
Shin (2026) [68]	+	−	−	+	−	−
Li M (2021) [69]	−	±	−	+	−	−
Kan (2026) [70]	+	+	+	+	−	−
Zhu (2021) [71]	+	+	+	+	−	−
Asadi (2024) [72]	+	+	−	−	−	−
Mousazadeh (2023) [73]	+	±	±	+	−	−
Atkinson (2025) [74]	−	−	+	−	−	−
Thabet (2022) [75]	±	−	−	−	−	−
Zhang H (2025) [76]	+	±	±	+	−	+
Aishajiang (2025) [77]	+	±	+	+	−	−
Shi (2024) [78]	+	±	±	+	−	−
Mehrnia (2021) [79]	+	−	−	−	−	−
Nosrati (2021) [80]	+	±	+	+	+	−
Nosrati (2022) [81]	+	+	+	+	−	−
Zhao (2016) [81]	+	+	+	+	−	−
Talik (2020) [82]	+	−	−	−	−	+
Colak (2024) [83]	−	−	±	+	−	−

Notes: Mechanistic and outcome classifications summarize the findings reported in the original studies. Biological effects such as increased ROS generation, DNA damage, apoptosis, immune activation, or tumor growth inhibition indicate radiation-associated biological enhancement (radioenhancement) but do not, by themselves, establish formal radiosensitization. According to radiobiological principles, formal radiosensitization requires quantitative validation using clonogenic survival assays, sensitizer enhancement ratio (SER), dose enhancement factor (DEF), dose-modifying factor (DMF), or equivalent dose–response analyses. ↓ Viability = reduced cell viability or clonogenic survival; ↑ Apoptosis = increased apoptotic markers (Annexin V, TUNEL, caspase activation); ↑ DNA Damage = γ-H2AX, comet assay, or repair inhibition; Tumor Growth ↓ = reduced tumor volume/weight in vivo; Survival ↑ = improved survival endpoints in animal models; SER/DEF = sensitizer enhancement ratio or dose enhancement factor; “±” = partial or indirect evidence; “–” = not reported.

## Data Availability

No new data were created or analyzed in this study. Data sharing is not applicable to this article.

## Supplementary Materials

The following supporting information can be downloaded at: https://www.mdpi.com/article/10.3390/ijms27146522/s1.

## Abbreviations

The following abbreviations are used in this manuscript:

AgNP	Silver nanoparticle
AuNP	Gold nanoparticle
BDP	Boron dipyrromethene
BSA	Bovine serum albumin
CAF	Cancer-associated fibroblast
cGAS	Cyclic GMP–AMP synthase
CUR	Curcumin
CXCR4	C-X-C chemokine receptor type 4
DEF	Dose enhancement factor
DMBA	7,12-Dimethylbenz[a]anthracene
DNA	Deoxyribonucleic acid
DOX	Doxorubicin
EMT	Epithelial–mesenchymal transition
FA	Folic acid
GOx	Glucose oxidase
GSH	Glutathione
HfO_2_	Hafnium oxide
HER2	Human epidermal growth factor receptor 2
*hNIS*	Human sodium iodide symporter
ICD	Immunogenic cell death
ICG	Indocyanine green
IV	In vitro
IVV	In vivo
MnO_2_	Manganese dioxide
MOF	Metal–organic framework
NP	Nanoparticle
OMV	Outer membrane vesicle
PBS	Phosphate-buffered saline
PDT	Photodynamic therapy
PEG	Polyethylene glycol
PEoz	Poly(2-ethyl-2-oxazoline)
PMAO	Poly(maleic anhydride-alt-1-octadecene)
PVP	Polyvinylpyrrolidone
QD	Quantum dot
RBC	Red blood cell
RCD	Regulated cell death
RGD	Arginine-glycine-aspartic acid peptide
RNAi	RNA interference
ROS	Reactive oxygen species
SAHA	Suberoylanilide hydroxamic acid
SER	Sensitizer enhancement ratio
siRNA	Small interfering RNA
STING	Stimulator of interferon genes
TNBC	Triple-negative breast cancer
TPP	Targeting peptide
γ-H2AX	Phosphorylated histone H2AX
