# Supplementary material for: Nanoparticle-Mediated Radiosensitization in Breast Cancer: A Systematic Review of Preclinical Evidence and Translational Challenges
Source: Int J Mol Sci. 2026 Jul 22;27(14):6522. doi: 10.3390/ijms27146522 (PMC13411440; doi:10.3390/ijms27146522)
Supplement: Supplementary file 1 [file ijms-27-06522-s001.zip › ijms-4373372-supplementary/Supplementary Table S1 Search.pdf]

Supplementary Table S1. Detailed Search Strategies

Database	Search Strategy
**MEDLINE (PubMed)**	("Breast Neoplasms"[Mesh] OR breast cancer*[tiab] OR breast neoplasm*[tiab] OR breast tumor*[tiab] OR mammary carcinoma*[tiab]) AND ("Radiotherapy"[Mesh] OR radiotherap*[tiab] OR irradiation[tiab] OR irradiat*[tiab] OR radiosensiti*[tiab] OR radiosensitizer*[tiab] OR dose enhancement[tiab] OR sensitizer enhancement ratio[tiab]) AND ("Nanoparticles"[Mesh] OR "Nanomedicine"[Mesh] OR nanoparticle*[tiab] OR nanomedicine[tiab] OR nanotechnology[tiab] OR nanocarrier*[tiab] OR nanomaterial*[tiab] OR liposom*[tiab] OR micelle*[tiab] OR "gold nanoparticle*"[tiab] OR "silver nanoparticle*"[tiab] OR "polymeric nanoparticle*"[tiab] OR "metal nanoparticle*"[tiab]) NOT (review[pt] OR systematic review[ti] OR meta-analysis[pt] OR editorial[pt] OR letter[pt]) AND (("2015/01/01"[Date - Publication] : "2026/03/02"[Date - Publication])) AND (English[lang])
**EMBASE (Elsevier Embase.com)**	('breast cancer'/exp OR breast cancer OR (breast AND cancer) OR 'breast tumor'/exp OR breast tumor OR (breast AND tumor)) AND ('radiotherapy'/exp OR radiotherapy OR irradiation OR irradiat* OR 'radiosensitizer'/exp OR radiosensitizer OR radiosensitization OR 'dose enhancement' OR 'sensitizer enhancement ratio') AND ('nanoparticle'/exp OR nanoparticle* OR 'nanocarrier'/exp OR nanocarrier* OR nanomedicine OR nanotechnology OR 'liposome'/exp OR liposome* OR micelle* OR 'gold nanoparticle'/exp OR 'silver nanoparticle'/exp OR 'metal nanoparticle') AND [2015-2026]/py AND [english]/lim AND ('in vitro study'/de OR 'in vivo study'/de OR 'animal experiment'/de OR 'cell culture'/de)
