# Supplementary material for: Nanoparticle-Mediated Radiosensitization in Breast Cancer: A Systematic Review of Preclinical Evidence and Translational Challenges
Source: Int J Mol Sci. 2026 Jul 22;27(14):6522. doi: 10.3390/ijms27146522 (PMC13411440; doi:10.3390/ijms27146522)
Supplement: Supplementary file 1 [file ijms-27-06522-s001.zip › ijms-4373372-supplementary/Supplementary Table S2 Quality.pdf]

Supplementary Table S2. Quality Assessment of Included Studies

Study	NP characterization	Model relevance	RT reporting	Outcome rigor	Mechanistic depth	Internal validity	Overall
Sun (2022) [15]	High	High	Moderate	High	Moderate	Moderate	High
Liu (2023) [16]	High	High	Moderate	High	Moderate	Moderate	High
Hu (2024) [17]	High	High	Moderate	High	High	Moderate	High
Shao (2024) [19]	High	Moderate	Moderate	High	Moderate	Moderate	High
Bhattarai (2021) [20]	High	High	Moderate	High	Moderate	Moderate	High
Wang X (2024) [21]	High	High	High	High	High	Moderate	High
Chen (2025) [22]	High	High	Moderate	High	High	Moderate	High
Samani (2020) [24]	Moderate	Moderate	Low	Moderate	Low	Moderate	Moderate
Cui (2017) [27]	Moderate	Moderate	Low	Moderate	Low	Low	Moderate
Nicol (2018) [28]	Moderate	Moderate	Low	Moderate	Low	Low	Moderate
Abdollahi (2023) [29]	Moderate	Moderate	Low	Moderate	Low	Moderate	Moderate
Swanner (2015) [30]	Moderate	Moderate	Low	Moderate	Low	Low	Moderate
Montazersaheb (2024) [31]	High	Moderate	Moderate	Moderate	Moderate	Moderate	Moderate
Zhang F (2023) [32]	High	Moderate	Moderate	High	Moderate	Moderate	High
Rashidzadeh (2023) [33]	High	Moderate	Moderate	Moderate	Moderate	Moderate	Moderate
Deng (2018) [34]	Moderate	Moderate	Low	Moderate	Low	Low	Moderate
Dastgir (2026) [35]	High	Moderate	Moderate	High	Moderate	Moderate	High
Yu (2023) [36]	High	Moderate	Moderate	High	Moderate	Moderate	High
Nosrati (2023) [37]	High	Moderate	Moderate	High	Moderate	Moderate	High
Wu (2023) [38]	High	Moderate	Moderate	High	Moderate	Moderate	High
Xiao (2023) [39]	High	High	Moderate	High	High	Moderate	High
Wang Y (2025) [40]	High	High	High	High	High	Moderate	High
Zhang J (2025) [41]	High	Moderate	Moderate	High	Moderate	Moderate	High
Minafra (2019) [42]	Moderate	Moderate	Low	Moderate	Low	Low	Moderate
Liu TI (2020) [43]	High	Moderate	Moderate	High	Moderate	Moderate	High
Chen (2024) [44]	High	Moderate	Moderate	Moderate	Moderate	Moderate	Moderate
Yang (2026) [45]	High	Moderate	Moderate	High	Moderate	Moderate	High
Bromma (2019) [46]	Moderate	Moderate	Low	Moderate	Low	Low	Moderate
Li P (2026) [47]	High	Moderate	Moderate	Moderate	Moderate	Moderate	Moderate
Karabuga (2023) [48]	High	Moderate	Moderate	Moderate	Moderate	Moderate	Moderate
Askar (2021) [49]	Moderate	Moderate	Low	Moderate	Low	Moderate	Moderate
Zhang Y (2026) [50]	High	Moderate	Moderate	Moderate	Moderate	Moderate	Moderate
Yamaguchi (2018) [51]	Moderate	Moderate	Low	Moderate	Low	Low	Moderate
Zetrini (2024) [52]	High	Moderate	Moderate	High	Moderate	Moderate	High
Abbasi (2016) [53]	Moderate	Moderate	Low	Moderate	Low	Low	Moderate

Study	NP characterization	Model relevance	RT reporting	Outcome rigor	Mechanistic depth	Internal validity	Overall
Nosrati (2022) [54]	High	Moderate	Moderate	High	Moderate	Moderate	High
Ghaffarlou (2023) [55]	High	Moderate	Moderate	High	Moderate	Moderate	High
Wang D (2024) [56]	High	High	High	High	Very high	Moderate	High
Musielak (2023) [57]	Moderate	Moderate	Low	Moderate	Low	Moderate	Moderate
Albers (2025) [58]	Low	Low	Low	Low	Low	Low	Low
Shiridokht (2025) [59]	Moderate	Moderate	Low	Moderate	Low	Moderate	Moderate
Hussein (2025) [60]	High	Moderate	Moderate	High	Moderate	Moderate	High
Zhang L (2021) [61]	Moderate	Moderate	Low	Moderate	Low	Moderate	Moderate
Cline (2021) [62]	Moderate	Moderate	Low	Moderate	Low	Moderate	Moderate
Mulgaonkar (2017) [63]	Moderate	Moderate	Low	Moderate	Low	Low	Moderate
Ghahremani (2018) [64]	Moderate	Moderate	Low	Moderate	Low	Low	Moderate
Kefayat (2019) [65]	Moderate	Moderate	Low	Moderate	Low	Low	Moderate
Detappe (2020) [66]	Moderate	Moderate	Low	Moderate	Low	Moderate	Moderate
Rahmani (2025) [67]	High	Moderate	Moderate	High	Moderate	Moderate	High
Shin (2026) [68]	High	Moderate	Moderate	High	High	Moderate	High
Li M (2021) [69]	Moderate	Moderate	Low	Moderate	Low	Moderate	Moderate
Kan (2026) [70]	High	Moderate	Moderate	High	Moderate	Moderate	High
Zhu (2021) [71]	High	Moderate	Moderate	High	Moderate	Moderate	High
Asadi (2024) [72]	Moderate	Moderate	Low	Moderate	Low	Moderate	Moderate
Mousazadeh (2023) [73]	High	Moderate	Moderate	High	Moderate	Moderate	High
Atkinson (2025) [74]	Moderate	Moderate	Low	Moderate	Low	Moderate	Moderate
Thabet (2022) [75]	Low	Low	Low	Low	Low	Low	Low
Zhang H (2025) [76]	High	Moderate	Moderate	High	Moderate	Moderate	High
Aishajiang (2025) [77]	High	Moderate	Moderate	High	High	Moderate	High
Shi (2024) [78]	High	Moderate	Moderate	High	Moderate	Moderate	High
Mehrnia (2021) [79]	Moderate	Moderate	Low	Moderate	Low	Moderate	Moderate
Nosrati (2021) [80]	High	Moderate	Moderate	High	Moderate	Moderate	High
Nosrati (2022) [81]	High	Moderate	Moderate	High	Moderate	Moderate	High
Zhao (2016) [82]	Moderate	Moderate	Low	Moderate	Low	Low	Moderate
Talik (2020) [83]	Moderate	Moderate	Low	Moderate	Low	Moderate	Moderate
Colak (2024) [84]	Moderate	Moderate	Low	Moderate	Low	Moderate	Moderate
