# Supplementary material for: Nanoparticle-Mediated Radiosensitization in Breast Cancer: A Systematic Review of Preclinical Evidence and Translational Challenges
Source: Int J Mol Sci. 2026 Jul 22;27(14):6522. doi: 10.3390/ijms27146522 (PMC13411440; doi:10.3390/ijms27146522)
Supplement: Supplementary file 1 [file ijms-27-06522-s001.zip › ijms-4373372-supplementary/Supplementary Table S3 Experimental Models and Nanoplatform Characteristics.pdf]

Supplementary Table S3. Detailed Experimental Models and Nanoplatform Characteristics

Study	Full Breast Cancer Model	Study Setting	Nanoparticle / Platform	Functionalization / Payload	Main RT Context
Sun (2022) [15]	Triple-negative breast cancer cell lines	In vitro	AGuIX nanoparticles	Ultrasmall gadolinium-based nanoparticles	X-ray RT
Liu (2023) [16]	Triple-negative breast cancer models	In vitro + in vivo	Polymeric nanoparticles	Epigenetic drug-loaded nanoplatform	X-ray RT
Hu (2024) [17]	4T1 cells; primary and distant tumor model	In vitro + in vivo	HfO_2@MnO_2@GOx	GOx-doped MnO_2-coated HfO_2	6 Gy localized X-ray RT
Shao (2024) [19]	Breast cancer models	In vitro + in vivo	Liposomal nanoplatform	GOx and MnO_2 co-loaded system	X-ray RT
Bhattarai (2021) [20]	Breast cancer models	In vitro + in vivo	Gold nanoparticles	CXCR4-targeting peptide	X-ray RT
Wang X (2024) [21]	Triple-negative breast cancer models	In vitro + in vivo	Oxygen-generating nanoplatform	Catalase-like hypoxia-modulating system	X-ray RT
Chen (2025) [22]	Metastatic TNBC models	In vitro + in vivo	CeO_2 nanobooster	OMV-modified nanoparticles	X-ray RT
Samani (2020) [24]	HER2-positive cells	In vitro	Gold nanoclusters	Trastuzumab + folic acid targeting	X-ray RT
Cui (2017) [27]	TNBC models	In vitro + in vivo	Gold nanoparticles	Cisplatin-combined system	X-ray RT
Nicol (2018) [28]	MCF-7 and MDA-MB-231	In vitro + in vivo	Functionalized gold nanoparticles	PEG + peptide targeting	X-ray RT
Abdollahi (2023) [29]	HER2-positive cells	In vitro	Fe_3O_4@Au nanoparticles	HER2-targeted core-shell	X-ray RT
Swanner (2015) [30]	TNBC and non-TNBC models	In vitro + in vivo	Silver nanoparticles	PVP-coated	X-ray RT
Montazersaheb (2024) [31]	TNBC cell lines	In vitro	Silver nanoparticles	Green-synthesized system	X-ray RT
Zhang F (2023) [32]	Breast cancer models	In vitro + in vivo	Platinum nanoassembly	Pt coordination nanostructure	X-ray RT
Rashidzadeh (2023) [33]	Breast tumor models	In vitro + in vivo	Platinum nanoparticles	Alginate-coated	X-ray RT
Deng (2018) [34]	4T1 murine model	In vitro + in vivo	Bismuth nanoparticles	RBC membrane coating	X-ray RT
Dastgir (2026) [35]	HER2-positive models	In vitro + in vivo	Bi_2O_3 nanoparticles	Chitosan/ALA/curcumin systems	X-ray RT
Yu (2023) [36]	TNBC models	In vitro + in vivo	Gd_2O_3 nanoparticles	Immune-activating system	X-ray RT
Nosrati (2023) [37]	Breast cancer models	In vitro + in vivo	Gd_2O_3/Au hybrid	BSA-capped hybrid	X-ray RT
Wu (2023) [38]	TNBC cell lines	In vitro	Fe_3O_4-Au hybrid	Hsp70-targeting peptide	X-ray RT

Study	Full Breast Cancer Model	Study Setting	Nanoparticle / Platform	Functionalization / Payload	Main RT Context
Xiao (2023) [39]	4T1 cells and mice	In vitro + in vivo	Au@$AgBiS_2$-PEG	PEG-modified core-shell	X-ray RT
Wang Y (2025) [40]	4T1 cells and mice	In vitro + in vivo	Pt@Ce-MOF-RGD/FA	Cisplatin-loaded MOF	RT
Zhang J (2025) [41]	4T1 cells and mice	In vitro + in vivo	MOs-G@DOX	Gd-loaded mesoporous system	4 Gy RT
Minafra (2019) [42]	Human cell lines	In vitro	Solid lipid nanoparticles	Curcumin-loaded	X-ray RT
Liu TI (2020) [43]	TNBC models	In vitro + in vivo	Polymeric nanoparticles	SAHA + ICG co-loaded	X-ray RT
Chen (2024) [44]	4T1 spheroids and model	In vitro + in vivo	ECM/Tel	Membrane-coated nanoparticles	RT
Yang (2026) [45]	TNBC models	In vitro + in vivo	RNAi nanoplatform	αTrop2 siRNA system	X-ray RT
Bromma (2019) [46]	Cell lines	In vitro	Lipid nanoparticle system	Gold delivery system	X-ray RT
Li (2026a) [47]	4T1 tumor-bearing mice	In vivo	Lipid nanoparticles	Sunitinib-loaded PEG system	Two-stage RT
Karabuga (2023) [48]	4T1 mice	In vivo	Liposomal radiosensitizer	QD–Ce6 conjugate	RT + PDT
Askar (2021) [49]	Cell lines	In vitro	MgO nanoparticles	HA + FA targeting	X-ray RT
Zhang Y (2026) [50]	Breast cancer models	In vitro + in vivo	Platinum nanoparticles	BSA-coated	X-ray RT
Yamaguchi (2018) [51]	SK-BR3 cells	In vitro	Silica nanoparticles	Anti-HER2 conjugation	X-ray RT
Zetrini (2024) [52]	TNBC models	In vitro + in vivo	siRNA nanoparticles	RAD50-targeting	X-ray RT
Abbasi (2016) [53]	EMT6 and MDA-MB-231	In vitro + in vivo	MnO_2 nanoparticles	Hypoxia-responsive system	X-ray RT
Nosrati (2022) [54]	4T1 model	In vitro + in vivo	Fe_3O_4/Bi_2S_3 nanoparticles	FA-functionalized	X-ray RT
Ghaffarlou (2023) [55]	4T1 models	In vitro + in vivo	Ag-Ag_2S@BSA-FA	FA functionalization	X-ray RT
Wang D (2024) [56]	4T1 model	In vitro + in vivo	Au/MnO_2 nanoparticles	Membrane-coated + siRNA	X-ray RT
Musielak (2023) [57]	MCF-7 cells	In vitro	Gold nanoparticles	Size-dependent system	X-ray RT
Albers (2025) [58]	Mouse model	In vivo	$BaSO_4$ nanoparticles	None	External beam RT
Shiridokht (2025) [59]	MCF-7 cells	In vitro	Ag + chitosan nanoparticles	Metformin-loaded system	X-ray RT
Hussein (2025) [60]	MCF-7 + DMBA model	In vitro + in vivo	Res-Ch-NPs	Resveratrol-loaded	γ-irradiation
Zhang L (2021) [61]	TNBC models	In vitro + in vivo	Au nanoclusters + gene system	hNIS delivery	^{131}I therapy

Study	Full Breast Cancer Model	Study Setting	Nanoparticle / Platform	Functionalization / Payload	Main RT Context
Cline (2021) [62]	Tumor models	In vitro + in vivo	KI nanoparticles	PMAO coating	I-131 RT
Mulgaonkar (2017) [63]	Xenograft model	In vivo	Hollow gold nanoparticles	None	X-ray RT
Ghahremani (2018) [64]	4T1 cells	In vitro	Gold nanoclusters	AS1411 aptamer	X-ray RT
Kefayat (2019) [65]	4T1 mice	In vivo	BSA-GNPs	FA/glucose decoration	MV RT
Detappe (2020) [66]	E0771 model	In vivo	Gadolinium nanoparticles	Anti-MUC1 antibody	X-ray RT
Rahmani (2025) [67]	MDA-MB-231	In vitro	Cur-Fe_3O_4@ZIF-8	Curcumin-loaded	2 Gy RT
Shin (2026) [68]	4T1 model	In vitro + in vivo	Lipid nanoparticles	siGPX4 system	X-ray RT
Li (2021) [69]	Orthotopic model	In vivo	GNPs + curcumin	Glucose tagging	X-ray RT
Kan (2026) [70]	TNBC models	In vitro + in vivo	Silver nanoclusters	Aptamer-functionalized	X-ray RT
Zhu (2021) [71]	Breast cancer models	In vitro + in vivo	Polymersomes	SAHA-loaded	X-ray RT
Asadi (2024) [72]	TNBC cells	In vitro	Zn nanoparticles	DOX-conjugated	X-ray RT
Mousazadeh (2023) [73]	Tumor models	In vitro + in vivo	Ag_2S nanoparticles	Alginate-coated	X-ray RT
Atkinson (2025) [74]	4T1 cells	In vitro	Transferrin-AuNPs	PEGylated targeting	X-ray RT
Thabet (2022) [75]	Cell lines	In vitro	Nanocomposite	Metabolic targeting	X-ray RT
Zhang H (2025) [76]	Breast cancer models	In vitro + in vivo	(as reported)	(as reported)	X-ray RT
Aishajiang (2025) [77]	4T1 model	In vitro + in vivo	Bi_2Se_3 nanomedicine	RSL3 + diABZi	X-ray RT
Shi (2024) [78]	Breast cancer models	In vitro + in vivo	BSNPs	Sorafenib-loaded	X-ray RT
Mehrnia (2021) [79]	Cell lines	In vitro	Gold nanoparticles	AS1411 aptamer	X-ray RT
Nosrati (2021) [80]	4T1 model	In vitro + in vivo	Fe_3O_4-Au nanoparticles	FA + curcumin	X-ray RT
Nosrati (2022) [81]	Breast cancer models	In vitro + in vivo	Polymeric nanoparticles	Gold prodrug-loaded	X-ray RT
Zhao (2016) [82]	TNBC cell lines	In vitro + in vivo	Gold nanorods	PEG + RGD	MV RT
Talik (2020) [83]	MCF-7 / MDA-MB-231	In vitro	Bi_2O_3 nanoparticles	Drug combination	X-ray RT
Colak (2024) [84]	Tumor-bearing model	In vivo	Bi_2S_3 nanoparticles	Hydrogel system	X-ray RT

Notes: TNBC = triple-negative breast cancer; HER2 = human epidermal growth factor receptor 2; RT = radiotherapy; IV = in vitro; IVV = in vivo; NP = nanoparticle; Au = gold; Ag = silver; Pt = platinum; Bi = bismuth; Gd = gadolinium; HfO_2 = hafnium oxide; MnO_2 = manganese dioxide; GOx = glucose oxidase; CeO_2 = cerium oxide; Fe_3O_4 = magnetite; Bi_2O_3 = bismuth oxide; Gd_2O_3 = gadolinium oxide; Ag_2S = silver sulfide; Bi_2Se_3 = bismuth selenide; MOF = metal–organic framework; PEG = polyethylene glycol; RGD = arginine-glycine-aspartic acid peptide; FA = folic acid; BSA = bovine serum albumin; RBC = red blood cell; OMV = outer membrane vesicle; PVP = polyvinylpyrrolidone; CUR = curcumin; DOX = doxorubicin; SAHA = suberoylanilide hydroxamic acid; ICG = indocyanine green; RNAi = RNA interference; siRNA = small interfering RNA; hNIS = human sodium iodide symporter; KI = potassium iodide; PMAO = poly(maleic anhydride-alt-1-octadecene); HA = hyaluronic acid; QD = quantum dot; Ce6 = chlorin e6; PDT = photodynamic therapy; ECM/Tel = erythrocyte membrane-coated telmisartan nanoparticles; Res-Ch-NPs = resveratrol-loaded chitosan nanoparticles; GNPs = gold nanoparticles; MV RT = megavoltage radiotherapy; γ-irradiation = gamma irradiation; RSL3 = ferroptosis inducer RSL3; diABZi = diamidobenzimidazole STING agonist; BSNPs = BDP-SS-PEG nanoparticles; BDP-SS-PEG = boron dipyrromethene-disulfide-polyethylene glycol; DMBA = 7,12-dimethylbenz[a]anthracene; MUC1 = mucin 1; SER = sensitizer enhancement ratio; DEF = dose enhancement factor.
