# Supplementary material for: Nanoparticle-Mediated Radiosensitization in Breast Cancer: A Systematic Review of Preclinical Evidence and Translational Challenges
Source: Int J Mol Sci. 2026 Jul 22;27(14):6522. doi: 10.3390/ijms27146522 (PMC13411440; doi:10.3390/ijms27146522)
Supplement: Supplementary file 1 [file ijms-27-06522-s001.zip › ijms-4373372-supplementary/Supplementary Table S4 Experimental Design and Radiation Parameters.pdf]

Supplementary Table S4. Experimental Design and Radiation Parameters

Study	In Vitro	In Vivo	Main Cell Line(s)	Animal Model	Radiation Type	Dose / Regimen
Sun (2022) [15]	+	–	TNBC cells	–	X-ray	Not specified
Liu (2023) [16]	+	+	TNBC cells	Tumor-bearing mice	X-ray	Not specified
Hu (2024) [17]	+	+	4T1	Primary + distant tumor model	X-ray	6 Gy localized RT
Shao (2024) [19]	+	+	Breast cancer cells	Tumor-bearing mice	X-ray	Not specified
Bhattarai (2021) [20]	+	+	Breast cancer cells	Tumor-bearing mice	X-ray	Not specified
Wang X (2024) [21]	+	+	TNBC cells	Tumor-bearing mice	X-ray	Not specified
Chen (2025) [22]	+	+	TNBC cells	Metastatic model	X-ray	Not specified
Samani (2020) [24]	+	–	HER2+ cells	–	X-ray	Not specified
Cui (2017) [27]	+	+	TNBC cells	Tumor-bearing mice	X-ray	Not specified
Nicol (2018) [28]	+	+	MCF-7, MDA-MB-231	Tumor model	X-ray	Not specified
Abdollahi (2023) [29]	+	–	HER2+ cells	–	X-ray	Not specified
Swanner (2015) [30]	+	+	TNBC cells	Tumor-bearing mice	X-ray	Not specified
Montazersaheb (2024) [31]	+	–	TNBC cells	–	X-ray	Not specified
Zhang F (2023) [32]	+	+	Breast cancer cells	Tumor-bearing mice	X-ray	Not specified
Rashidzadeh (2023) [33]	+	+	Breast cancer cells	Tumor-bearing mice	X-ray	Not specified
Deng (2018) [34]	+	+	4T1	4T1 murine model	X-ray	Not specified
Dastgir (2026) [35]	+	+	HER2+ cells	Tumor model	X-ray	Not specified
Yu (2023) [36]	+	+	TNBC cells	Tumor-bearing mice	X-ray	Not specified
Nosrati (2023) [37]	+	+	Breast cancer cells	Tumor-bearing mice	X-ray	Not specified
Wu (2023) [38]	+	–	TNBC cells	–	X-ray	Not specified
Xiao (2023) [39]	+	+	4T1	BALB/c model	X-ray	~6 Gy
Wang Y (2025) [40]	+	+	4T1	4T1 tumor model	RT	Not specified
Zhang J (2025) [41]	+	+	4T1	Tumor-bearing mice	X-ray	4 Gy
Minafra (2019) [42]	+	–	Breast cancer cells	–	X-ray	Not specified
Liu TI (2020) [43]	+	+	TNBC cells	Tumor model	X-ray	Not specified
Chen (2024) [44]	+	+	4T1 spheroids	Orthotopic model	RT	Not specified
Yang (2026) [45]	+	+	TNBC cells	Tumor-bearing mice	X-ray	Not specified
Bromma (2019) [46]	+	–	Breast cancer cells	–	X-ray	Not specified
Li (2026a) [47]	–	+	–	4T1	RT	Two-stage RT
Karabuga (2023) [48]	–	+	–	4T1	RT + PDT	Not specified
Askar (2021) [49]	+	–	Breast cancer cells	–	X-ray	Not specified
Zhang Y (2026) [50]	+	+	Breast cancer cells	Tumor-bearing mice	X-ray	Not specified
Yamaguchi (2018) [51]	+	–	SK-BR3	–	X-ray	Not specified
Zetrini (2024) [52]	+	+	TNBC cells	TNBC model	X-ray	Not specified
Abbasi (2016) [53]	+	+	EMT6, MDA-MB-231	EMT6 model	X-ray	Not specified

Study	In Vitro	In Vivo	Main Cell Line(s)	Animal Model	Radiation Type	Dose / Regimen
Nosrati (2022) [54]	+	+	4T1	4T1 model	X-ray	Not specified
Ghaffarlou (2023) [55]	+	+	4T1	4T1 model	X-ray	Not specified
Wang D (2024) [56]	+	+	4T1	4T1 model	X-ray	Not specified
Musielak (2023) [57]	+	–	MCF-7	–	X-ray	Not specified
Albers (2025) [58]	–	+	–	Basal-like model	External beam RT	Not specified
Shiridokht (2025) [59]	+	–	MCF-7	–	X-ray	Not specified
Hussein (2025) [60]	+	+	MCF-7	DMBA model	Gamma RT	Not specified
Zhang L (2021) [61]	+	+	TNBC cells	Tumor-bearing mice	I-131	Not specified
Cline (2021) [62]	+	+	Breast cancer cells	Tumor-bearing mice	I-131	Not specified
Mulgaonkar (2017) [63]	–	+	–	Xenograft	X-ray	Not specified
Ghahremani (2018) [64]	+	–	4T1	–	X-ray	Not specified
Kefayat (2019) [65]	–	+	–	4T1 BALB/c	MV RT	6 Gy
Detappe (2020) [66]	–	+	–	E0771 model	X-ray	Not specified
Rahmani (2025) [67]	+	–	MDA-MB-231	–	X-ray	2 Gy
Shin (2026) [68]	+	+	4T1	4T1 model	X-ray	Not specified
Li (2021) [69]	–	+	–	Xenograft	X-ray	Not specified
Kan (2026) [70]	+	+	TNBC cells	Tumor-bearing mice	X-ray	Not specified
Zhu (2021) [71]	+	+	Breast cancer cells	Tumor-bearing mice	X-ray	Not specified
Asadi (2024) [72]	+	–	TNBC cells	–	X-ray	Not specified
Mousazadeh (2023) [73]	+	+	Breast cancer cells	Tumor model	X-ray	Not specified
Atkinson (2025) [74]	+	–	4T1	–	X-ray	~1.9 Gy
Thabet (2022) [75]	+	–	Breast cancer cells	–	X-ray	Not specified
Zhang H (2025) [76]	+	+	Breast cancer cells	Tumor-bearing mice	X-ray	Not specified
Aishajiang (2025) [77]	+	+	4T1	4T1 model	X-ray	Not specified
Shi (2024) [78]	+	+	Breast cancer cells	Tumor-bearing mice	X-ray	Not specified
Mehrnia (2021) [79]	+	–	Breast cancer cells	–	X-ray	Not specified
Nosrati (2021) [80]	+	+	4T1	4T1 model	X-ray	Not specified
Nosrati (2022) [81]	+	+	Breast cancer cells	Tumor-bearing mice	X-ray	Not specified
Zhao (2016) [82]	+	+	TNBC cells	Tumor-bearing mice	MV X-ray	Not specified
Talik (2020) [83]	+	–	MCF-7, MDA-MB-231	–	X-ray	Not specified
Colak (2024) [84]	–	+	–	Breast tumor model	X-ray	Not specified

Notes: TNBC = triple-negative breast cancer; HER2+ = human epidermal growth factor receptor 2-positive; IV = in vitro; IVV = in vivo; RT = radiotherapy; PDT = photodynamic therapy; MV RT = megavoltage radiotherapy; MV X-ray = megavoltage X-ray irradiation; Gamma RT = gamma irradiation; GOx = glucose oxidase; OMV = outer membrane vesicle; PEG = polyethylene glycol; PVP = polyvinylpyrrolidone; RBC = red blood cell; BSA = bovine serum albumin; MOF = metal–organic framework; DMBA = 7,12-dimethylbenz[a]anthracene; I-131 = iodine-131 radionuclide therapy; MCF-7 = Michigan Cancer Foundation-7 breast cancer cell line; MDA-MB-231 = human triple-negative breast cancer

cell line; 4T1 = murine triple-negative breast cancer cell line; E0771 = murine breast adenocarcinoma model; SK-BR3 = HER2-positive human breast cancer cell line; “+” = present/reported; “–” = absent/not reported.
