# Supplementary material for: Nanoparticle-Mediated Radiosensitization in Breast Cancer: A Systematic Review of Preclinical Evidence and Translational Challenges
Source: Int J Mol Sci. 2026 Jul 22;27(14):6522. doi: 10.3390/ijms27146522 (PMC13411440; doi:10.3390/ijms27146522)
Supplement: Supplementary file 1 [file ijms-27-06522-s001.zip › ijms-4373372-supplementary/Supplementary Table S5 Physicochemical Characteristics of Nanoparticles.pdf]

Supplementary Table S5. Physicochemical Characteristics of Nanoparticles

Study	Nanoparticle Class	Size (nm)	Surface / Coating	Charge / Stability	Physicochemical Comment
Sun (2022) [15]	AGuIX NP	~3–5 nm	None	Highly stable	Ultrasmall radiosensitizer
Liu (2023) [16]	Polymeric NP	~100 nm	Drug-loaded polymer	Stable	Epigenetic modulation
Hu (2024) [17]	HfO_2@MnO_2@GOx	~100 nm	Multi-layer	Stable	Cascade catalytic system
Shao (2024) [19]	Liposome	~100–150 nm	Lipid bilayer	Stable	Enzyme-loaded nanoplatform
Bhattarai (2021) [20]	Au NP	~20–50 nm	Peptide-modified	Stable	Targeted radiosensitizer
Wang X (2024) [21]	Nanoplatform	~100 nm	Catalase-like	Stable	Oxygen-generating system
Chen (2025) [22]	CeO_2 NP	~50–100 nm	OMV-coated	Stable	Immune-activating
Samani (2020) [24]	Au nanocluster	<5 nm	Antibody + FA	Stable	HER2 targeting
Cui (2017) [27]	Au NP	~20–30 nm	Drug-conjugated	Stable	Chemo-radiosensitizer
Nicol (2018) [28]	Au NP	~13–50 nm	PEG + peptides	Stable	Enhanced uptake
Abdollahi (2023) [29]	Fe_3O_4@Au NP	~30–50 nm	HER2-targeted	Stable	Magnetic targeting
Swanner (2015) [30]	Ag NP	~10–30 nm	PVP-coated	Stable	Selective toxicity
Montazersaheb (2024) [31]	Ag NP	~10–30 nm	None	Stable	Intrinsic cytotoxicity
Zhang F (2023) [32]	Pt nanoassembly	~50–100 nm	Coordination complex	Stable	Mixed-valence Pt system
Rashidzadeh (2023) [33]	Pt NP	~20–50 nm	Alginate-coated	Stable	Biocompatible Pt system
Deng (2018) [34]	Bi NP	~50–80 nm	RBC membrane	Stable	Biomimetic NP
Dastgir (2026) [35]	Bi_2O_3 NP	~100 nm	Multi-functional coating	Stable	Multi-drug NP
Yu (2023) [36]	Gd_2O_3 NP	~20–50 nm	None	Stable	Immune modulation
Nosrati (2023) [37]	Hybrid NP	~30–50 nm	BSA coating	Stable	Gd/Au dual functionality
Wu (2023) [38]	Fe_3O_4-Au NP	~20–40 nm	Peptide-modified	Stable	Targeted magnetic NP
Xiao (2023) [39]	Core-shell NP	~100 nm	PEG-modified	Stable	High-Z composite
Wang Y (2025) [40]	MOF NP	~100 nm	Dual-targeted	Stable	Cisplatin carrier
Zhang J (2025) [41]	Organosilica NP	~250–280 nm	Gd + DOX-loaded	Stable	Theranostic NP
Minafra (2019) [42]	Solid lipid NP	~100–200 nm	Lipid matrix	Stable	Hydrophobic drug carrier
Liu TI (2020) [43]	Polymeric NP	~100 nm	ROS-responsive	Stable	Phototherapeutic NP
Chen (2024) [44]	Polymeric NP	~100 nm	RBC membrane	Stable	Biomimetic delivery
Yang (2026) [45]	RNA NP	~100 nm	Targeted polymer	Stable	Gene silencing
Bromma (2019) [46]	Lipid NP	~100 nm	Lipid-based	Stable	Carrier for Au NP
Li (2026a) [47]	Lipid NP	~100 nm	PEGylated	Stable	Drug delivery
Karabuga (2023) [48]	Liposome	~100 nm	PEG + FA	Stable	Photosensitizer carrier
Askar (2021) [49]	MgO NP	~50 nm	HA/FA coating	Stable	Dual-targeting system

Study	Nanoparticle Class	Size (nm)	Surface / Coating	Charge / Stability	Physicochemical Comment
Zhang Y (2026) [50]	Pt NP	~20–50 nm	BSA-coated	Stable	High-Z radiosensitizer
Yamaguchi (2018) [51]	Silica NP	~50 nm	HER2 antibody	Stable	Targeted delivery
Zetrini (2024) [52]	siRNA NP	~100 nm	Polymeric	Stable	Gene delivery vector
Abbasi (2016) [53]	MnO_2 NP	~50–100 nm	Bare MnO_2	Stable	H_2O_2-responsive oxygen generation
Nosrati (2022) [54]	Janus NP	~50–80 nm	BSA + FA	Stable	Dual-metal heterostructure
Ghaffarlou (2023) [55]	Ag-Ag_2S NP	~50–100 nm	BSA + FA	Stable	Hybrid NP
Wang D (2024) [56]	Hybrid NP	~100 nm	Cell membrane-coated	Stable	Biomimetic targeting
Musielak (2023) [57]	Au NP	Variable	None	Stable	Size-dependent effects
Albers (2025) [58]	$BaSO_4$ NP	~100 nm	None	Stable	High-density NP
Shiridokht (2025) [59]	Ag + chitosan NP	~100–200 nm	Chitosan-coated	Stable	Dual NP system
Hussein (2025) [60]	Chitosan NP	~150–200 nm	Resveratrol-loaded	Stable	Sustained release
Zhang L (2021) [61]	Au nanocluster	<5 nm	Gene-loaded	Stable	Gene delivery
Cline (2021) [62]	KI NP	Nano-range	PMAO coating	Stable	Iodide delivery via NIS
Mulgaonkar (2017) [63]	Hollow Au NP	~50 nm	None	Stable	Hollow structure
Ghahremani (2018) [64]	Au nanocluster	<5 nm	Aptamer + BSA	Stable	Targeted nanocluster
Kefayat (2019) [65]	Au NP	~20–30 nm	BSA + FA/glucose	Stable	Metabolic targeting
Detappe (2020) [66]	Gd NP	<5 nm	Antibody-conjugated	Stable	Targeted ultrasmall NP
Rahmani (2025) [67]	Fe_3O_4@ZIF-8	~100 nm	Curcumin-loaded	Stable	MOF-based delivery
Shin (2026) [68]	Lipid NP	~100 nm	Functional lipid	Stable	Ferroptosis-inducing
Li (2021) [69]	Au NP	~20–30 nm	Glucose-tagged	Stable	Metabolic targeting
Kan (2026) [70]	Nanocluster	<10 nm	Aptamer-modified	Stable	Ultrasmall targeting NP
Zhu (2021) [71]	Polymersome	~100 nm	Polymeric vesicle	Stable	Redox-sensitive drug release
Asadi (2024) [72]	Zn NP	~50–100 nm	Alginate-coated	Stable	Drug-conjugated NP
Mousazadeh (2023) [73]	Ag_2S NP	~50–100 nm	Alginate-coated	Stable	Biocompatible NP
Atkinson (2025) [74]	Au NP	~15–20 nm	PEG + transferrin	Stable	Targeted delivery
Thabet (2022) [75]	Nanocomposite	Nano-range	Composite	Stable	Metabolic targeting
Zhang H (2025) [76]	Liposome	~100–150 nm	RGD-modified	Stable	Dual-drug delivery
Aishajiang (2025) [77]	Bi_2Se_3 NP	~100 nm	Lipid-modified	Stable	Ferroptosis induction

Study	Nanoparticle Class	Size (nm)	Surface / Coating	Charge / Stability	Physicochemical Comment
Shi (2024) [78]	Polymeric NP	~150 nm	PEG polymer	Stable	GSH-responsive
Mehrnia (2021) [79]	Au NP	~20–30 nm	Aptamer-modified	Stable	Targeted cellular uptake
Nosrati (2021) [80]	Fe_3O_4-Au NP	~50 nm	BSA + FA + curcumin	Stable	Multifunctional NP
Nosrati (2022) [81]	Polymeric NP	~100 nm	Drug-loaded	Stable	Prodrug carrier
Zhao (2016) [82]	Au nanorods	~50 nm core	MSN + PEG + RGD	Stable	High-Z targeted system
Talik (2020) [83]	Bi_2O_3 NP	~50–100 nm	Drug-combined	Stable	Chemo combination
Colak (2024) [84]	Bi_2S_3 NP	~50–100 nm	Alginate hydrogel	Stable	Localized delivery

Notes: NP = nanoparticle; Au = gold; Ag = silver; Pt = platinum; Bi = bismuth; Gd = gadolinium; Zn = zinc; MgO = magnesium oxide; HfO_2 = hafnium oxide; MnO_2 = manganese dioxide; CeO_2 = cerium oxide; Fe_3O_4 = magnetite/iron oxide; Bi_2O_3 = bismuth oxide; Gd_2O_3 = gadolinium oxide; $BaSO_4$ = barium sulfate; KI = potassium iodide; Ag_2S = silver sulfide; Bi_2S_3 = bismuth sulfide; Bi_2Se_3 = bismuth selenide; ZIF-8 = zeolitic imidazolate framework-8; MOF = metal–organic framework; MSN = mesoporous silica nanoparticle; PEG = polyethylene glycol; FA = folic acid; HA = hyaluronic acid; RGD = arginine-glycine-aspartic acid peptide; BSA = bovine serum albumin; PVP = polyvinylpyrrolidone; RBC = red blood cell; OMV = outer membrane vesicle; PMAO = poly(maleic anhydride-alt-1-octadecene); DOX = doxorubicin; siRNA = small interfering RNA; GSH = glutathione; H_2O_2 = hydrogen peroxide; NIS = sodium/iodide symporter; HER2 = human epidermal growth factor receptor 2; GNP = gold nanoparticle; "nano-range" = nanoparticle size reported without precise numerical value.
